# Antioxidant and Antimicrobial Potential of Romanian Propolis Extract Combinations: Linking Chemical Composition with Biological Activity

**DOI:** 10.3390/antibiotics15100972

**Published:** 2026-10-01

**Authors:** Alexandru Ioan Giurgiu, Gianluca Albanese, Adriana Cristina Urcan, Victorița Bonta, Antonio De Cristofaro, Massimo Iorizzo, Otilia Bobiș, Daniel Severus Dezmirean

**Affiliations:** 1 Department of Apiculture and Sericulture, Faculty of Animal Science and Biotechnology, University of Agricultural Sciences and Veterinary Medicine Cluj-Napoca, 400372 Cluj-Napoca, Romania; alexandru.giurgiu@usamvcluj.ro (A.I.G.); victorita.bonta@usamvcluj.ro (V.B.); obobis@usamvcluj.ro (O.B.); ddezmirean@usamvcluj.ro (D.S.D.); 2 Department of Agriculture, Environmental and Food Sciences, University of Molise, Via De Sanctis snc, 86100 Campobasso, Italy; decrist@unimol.it (A.D.C.); iorizzo@unimol.it (M.I.); 3 Department of Microbiology and Immunology, Faculty of Animal Science and Biotechnology, University of Agricultural Sciences and Veterinary Medicine Cluj-Napoca, 400372 Cluj-Napoca, Romania

**Keywords:** propolis, Romanian propolis, phenolic compounds, antimicrobial activity, antioxidant activity, propolis combinations, synergistic interactions, multidimensional data analysis

## Abstract

**Background/Objectives**: Propolis is a chemically complex product whose antimicrobial activity varies with geographical origin and composition. This study evaluated the chemical, antioxidant, and antimicrobial properties of five Romanian propolis ethanolic extracts (PEEs) and their combinations, focusing on antimicrobial interactions and chemical–biological relationships. **Methods**: Five PEEs were evaluated individually, as ten binary combinations, and as one five-extract mixture. Antioxidant activity and 20 phenolic compounds were determined. Antimicrobial activity against 16 strains was assessed using agar diffusion and broth microdilution assays. Extract interactions were evaluated using the fractional inhibitory concentration index (FICI). Principal Component Analysis (PCA), Spearman correlation, and Partial Least Squares (PLS) regression investigated chemical–biological relationships. **Results:** Significant differences were observed among preparations in phenolic composition, antioxidant activity, and antimicrobial responses. Total phenolic content ranged from 88.8 to 149.8 mg GAE/g, while A + S showed the highest antioxidant activity (3213.74 mg TE/g). C1 showed strong activity against several Gram-positive bacteria and *Candida albicans*, whereas S and S-containing combinations showed activity against several Gram-negative strains. FICI analysis showed predominantly indifferent interactions (60.8%), followed by additive (25.0%), synergistic (9.09%), and antagonistic (5.11%). The strongest synergy was observed for A + S against *Pseudomonas aeruginosa* (FICI = 0.195). Chemical PC1 was inversely associated with biological PC2 (ρ = −0.809, FDR-adjusted *p* = 0.0013). **Conclusions**: Antimicrobial activity depends on extract composition and the target microorganism. Combining extracts did not consistently improve activity; however, selected combinations produced additive or synergistic interactions, whereas antagonism was uncommon. Multivariate analyses further supported an association between phenolic composition and antimicrobial variability.

## 1. Introduction

Propolis, or bee glue, is a complex natural resinous substance produced by *Apis mellifera* by mixing various plant exudates with wax and their saliva [1]. Honey bees use propolis to protect the colony from external threats such as pathogens or pests, but it is also used to seal small gaps or to glue structural elements; nonetheless, they use it as a vital structural and hygienic multi-tool [1,2,3]. Considering that some of the raw materials originate from the local flora available in the immediate hive area, it is well known that the properties of propolis can vary based on the compounds collected from the environment; as a result, the antimicrobial activities can also be variable [2,4]. Previous studies have demonstrated that the biological activity of propolis varies according to its geographical origin, with samples collected from different regions exhibiting distinct levels of activity [4,5,6]. Furthermore, the composition of propolis is not fixed, and on top of the chemical fingerprint given by the local flora, its composition can be influenced by the extraction method [4,7]. Given the propolis’ antimicrobial, antifungal and antiviral activity, it has been used since ancient times in human medicine [8,9]. It is known that ancient Egyptians implemented the use of propolis during the embalming process [4,10]. The Greeks and Romans documented its cicatrizant and antiseptic properties, while the Incas used it for fever reduction [10]. Due to its properties, propolis has been integrated even in modern pharmacology, and it can be found in cosmetics, nutraceuticals, foods and alternative medicine and has been intensively researched [1,3,9,10].

With the increasing concern regarding antibiotic resistance, propolis is a promising solution in the fight against antibiotic-resistant bacteria [8,11,12]. Some studies have shown the potential of propolis to stimulate, suppress, or help balance the immune response, depending on the dose and extract type. However, its effects remain dependent on the biological context, and a lack of standardisation still represents a limitation, although an ISO standard for raw propolis material specifications is available [13], as well as a standard for propolis extracts used in construction. Nevertheless, propolis extracts can be and are used in the development of new drugs and in the fight against infections [10]. Furthermore, in addition to the proven antimicrobial activity of propolis itself, research has highlighted its potential to exert synergistic effects when combined with conventional antibiotics [14,15,16].

Moreover, the concept of mixing propolis extracts from different areas and/or years has been explored. Despite the variability associated with botanical origin and other factors, blending propolis appears to attenuate differences in total phenolic and flavonoid content. Moreover, the antimicrobial and antioxidant bioactivity of the resulting mixtures appears to be maintained at a level similar to that of the most active extract in the mixture or, in some cases, enhanced [17]. Another potential benefit of mixing propolis from different sources is that it may contribute to the standardization process, which could facilitate its further applications in medicine [17,18]. It is known that many polyphenols are unstable and may undergo oxidation and degradation during storage or as a result of different processing techniques [19]. Their stability depends on the chemical structure of the compounds, the pH of the environment or solution, and storage temperature. When analysing changes in polyphenolic content, the determination of antioxidant activity is also an important approach for assessing the stability of polyphenols [20,21]. As demonstrated recently, the chemical structure of polyphenols is an important factor influencing their overall stability. The presence, number, and positioning of hydroxyl groups can also greatly influence their stability [22], with phenolic acids such as chlorogenic acids and their derivatives being more stable than certain flavonoids, including luteolin, quercitrin, and hesperetin [23].

The study included the characterisation of flavonoids and phenolic compounds, together with the assessment of antimicrobial activity, minimum inhibitory concentration (MIC), fractional inhibitory concentration (FIC), and antioxidant activity. Overall, 16 propolis preparations were evaluated, comprising five individual extracts, ten binary combinations, and one mixture containing all five extracts. Furthermore, the chemical composition of these preparations was examined in relation to their antioxidant and antimicrobial properties.

## 2. Materials and Methods

### 2.1. Propolis Samples Origin

In this study, five hydroalcoholic propolis extracts obtained between June and July 2024 from different regions of Romania were used. The samples were extracted in 80% ethanol and initially characterised for their chemical composition and biological properties as described by Albanese et al. [6]. Following extraction, the propolis ethanolic extracts (PEEs) were stored at room temperature in the dark. In the present study, the PEEs were evaluated after approximately two years of storage to assess whether they retained their original properties and whether combinations of extracts from different geographical locations exhibited effects distinct from those observed for the individual extracts.

### 2.2. Propolis Samples and Extract Combinations

The five original PEEs were used as individual extracts in the present study. In addition, all possible pairwise combinations of the five extracts were prepared by mixing equal volumes (1:1, *v*/*v*) of each extract, resulting in 10 combinations. Finally, a mixture containing equal volumes of all five extracts was also prepared. Overall, 16 samples were analysed, comprising five individual extracts, 10 pairwise combinations, and one mixture containing all five extracts.

The five individual propolis extracts and their combinations were prepared at 1% (*w*/*v*) for chemical characterisation and antioxidant analyses. A separate set of the same propolis extracts and combinations was prepared at 10% (*w*/*v*) exclusively for the antimicrobial assays described in Section 2.7 and Section 2.8.

### 2.3. Total Phenolics and Flavone/Flavonol Content

The total phenolic content (TPC) was determined using the Folin–Ciocalteu colourimetric method, following a procedure adapted from ISO2430:2023 [13]. Measurements were performed using a multichannel spectrophotometer (Synergy HT Multi-Mode, BioTek, Winooski, VT, USA). An aliquot of PEE (200 μL) was mixed with 1.5 mL ultrapure water, 400 μL Folin–Ciocalteu reagent, and 600 μL of 20% sodium carbonate solution. Ultrapure water was then added to a final volume of 5 mL. After incubation for 30 min at room temperature in the dark, absorbance was measured at 760 nm. A gallic acid calibration curve was constructed with concentrations of 1.0, 2.0, 3.0, 4.0, 5.0, 6.0, and 8.0 μg/mL. Results were expressed as mg of gallic acid equivalents (GAE)/g of propolis. All samples were analysed in triplicate.

For total flavone/flavonol content (TFC), a 25 mL volumetric flask was prepared with 1 mL PEE, 15 mL methanol, and 500 μL of 5% aluminium chloride solution. The volume was adjusted to 25 mL with methanol. The solution was incubated at room temperature in the dark for 30 min, and absorbance was measured at 425 nm. A quercetin calibration curve was constructed with final concentrations of 1.0, 2.0, 3.0, 4.0, 7.0, and 10.0 μg/mL. Results were expressed as mg of quercetin equivalents (QE)/g of propolis, and all samples were analysed in triplicate.

### 2.4. Antioxidant Activity Assays

#### 2.4.1. ABTS·+ Assay

The antioxidant activity of propolis ethanolic extracts (PEEs) was assessed using the ABTS·+ method as described by Mărgăoan et al. [24]. This assay measured the antioxidant capacity based on the reaction between the ABTS·+ radical cation and antioxidant compounds in the extracts. ABTS·+ was dissolved in pure methanol at a concentration of 7 mM. The radical cation was generated by reacting the methanolic ABTS solution with 2.45 mM potassium persulfate and incubating the mixture in the dark for 24 h. The resulting solution was diluted to an optical density of 0.700 at 734 nm prior to the assay. For the measurement, 30 μL of sample was added to 170 μL of ABTS·+ solution, and absorbance was recorded at 734 nm after 6 min in the dark using a spectrophotometer (BioTek Instruments, Winooski, VT, USA). Trolox served as the standard for the calibration curve, and antioxidant activity was expressed as Trolox equivalents per gram (Trolox Eq./g). Ascorbic acid was used as a positive control.

#### 2.4.2. DPPH·Assay

The activity of PEEs was also evaluated using the DPPH assay according to the method of Urcan et al. [25]. A total of 40 μL of ethanolic extract of propolis was added to 200 μL of DPPH solution (0.02 mg/mL). Ascorbic acid was used as a positive control. The absorbance of the samples was measured at 517 nm after 15 min using a spectrophotometer (BioTek Instruments, Winooski, VT, USA). Trolox was used as a standard for the calibration curve, and the antioxidant activity was expressed as Trolox Eq./g.

#### 2.4.3. FRAP Assay

The antioxidant activity of PEEs was evaluated using the ferric-reducing antioxidant power (FRAP) assay, as described by Urcan et al. [26]. In this method, 300 μL of FRAP reagent was mixed with 10 μL of sample and 10 μL of ultrapure water. The same procedure was applied to the positive control. After five minutes of incubation at 37 °C, absorbance was measured at 593 nm using a microplate reader (BioTek Instruments, Winooski, VT, USA). Trolox was used as the standard for the calibration curve, and antioxidant activity was expressed as Trolox equivalents per gram (Trolox Eq./g).

#### 2.4.4. ORAC Assay

The oxygen radical absorbance capacity (ORAC) of each individual extract and extract combination was determined using a hydrophilic ORAC assay adapted from Qiao et al. [27]. Briefly, 20 μL of diluted sample extract, phosphate buffer (pH 7.4) as the blank, or Trolox calibration solutions were added to a clear-bottom 96-well microplate. Subsequently, 150 μL of fluorescein solution (8.16 × 10^−2^ μmol/L) was added to each well, and the plate was incubated at 37 °C for 10 min. The reaction was initiated by adding 30 μL of 2,2′-Azobis-(2-amidinopropane) dihydrochloride (AAPH; 153 mmol/L), which acted as the peroxyl radical generator.

Fluorescence was measured at 1 min intervals for 50 min using a microplate reader (Synergy HT, BioTek Instruments, Inc., Winooski, VT, USA), with excitation and emission wavelengths of 485 nm and 528 nm, respectively. ORAC values were calculated using a linear regression model between Trolox concentration and the net area under the curve (AUC) of the fluorescein decay curve. The calibration range was 6.25–100 μmol/L Trolox. ORAC values were expressed as mg Trolox equivalents (TE)/g of propolis sample. The AUC was calculated using the following equation based on Zhang et al. [28].

AUC = 0.5 + f1/f_0_ + f_2_/f_0_ + … + f_49_/f_0_ + 0.5(f_50_/f_0_)

where f_0_ represents the initial fluorescence measured at 0 min, and f_50_ represents the fluorescence measured at 50 min. The net AUC value was calculated by subtracting the AUC of the blank from the AUC of each sample.

### 2.5. Chromatographic Conditions

The phenolic profile of propolis samples was determined according to the method described by Cucu et al. [29]. Samples were analysed by high-performance liquid chromatography coupled with a photodiode array detector (HPLC-PDA) using a Shimadzu LC-10ASD VY binary gradient system (Shimadzu Instruments, Kyoto, Japan), equipped with a controller, pumps, column oven, and autosampler. Separation was performed using a Teknokroma Mediterranean Sea 18 column (150 × 4.6 mm, 5 μm) (Teknokroma, Barcelona, Spain) at a flow rate of 1 mL/min. The binary gradient elution system consisted of (A) water adjusted to pH 2.5 with orthophosphoric acid and (B) acetonitrile. The gradient programme was as follows: 5% B at the beginning of the analysis, increasing to 9% B at 3 min, 13% B at 20 min, 33% B at 30 min, and 43% B from 42 to 60 min using a linear gradient. The proportion of B was subsequently increased to 90% at 65 min and to 100% at 70 min, followed by a gradual return to the initial conditions (5% B) until 80 min. The column temperature was maintained at 28 °C using the column oven. Detection was performed over the wavelength range of 220–400 nm [30]. HPLC analyses were performed using 1% propolis tinctures prepared in 80% ethanol. For the binary mixtures, equal volumes of the corresponding individual tinctures were combined without further dilution. The identity of each compound was confirmed by comparing its UV spectrum and retention time (Rt) with those of the corresponding analytical standard. The standards included protocatechuic acid, *p*-hydroxybenzoic acid, catechin, vanillic acid, chlorogenic acid, caffeic acid, vanillin, *p*-coumaric acid, ferulic acid, naringin, rosmarinic acid, quercitrin, quercetin, naringenin, apigenin, kaempferol, caffeic acid phenethyl ester (CAPE), chrysin, pinocembrin, and galangin. Individual compound standards (1000 mg/L) were prepared in HPLC-grade methanol. Calibration curves were prepared individually for each phenolic compound over the concentration range of 0.05–100 mg/L. The coefficients of determination (r^2^) for all calibration curves were >0.998. Each phenolic compound was quantified based on the corresponding peak area and calibration curve.

The limits of detection (LOD) and quantification (LOQ) were determined from six independent mixed standard solutions analysed under repeatability conditions. Measurements were performed at the lowest concentration producing a detectable chromatographic signal for each compound. The mean concentration (c) and standard deviation (SD) were calculated from the six replicate measurements. The LOD and LOQ were subsequently calculated as c + 3SD and c + 10SD, respectively. Results were expressed as μg/g of matrix (ppm).

### 2.6. Microbial Strains and Culture Conditions

The antimicrobial activity was evaluated against a panel of 16 reference microorganisms obtained from the American Type Culture Collection (ATCC), comprising six Gram-positive bacteria, eight Gram-negative bacteria, and two yeasts.

The Gram-positive panel included *Staphylococcus aureus* MRSA ATCC 43300, *S. aureus* ATCC 25923, *Staphylococcus epidermidis* ATCC 12228, *Enterococcus faecalis* ATCC 29212, *Enterococcus faecium* ATCC 19434, and *Listeria monocytogenes* ATCC 35152. The Gram-negative panel comprised *Escherichia coli* ATCC 8739, *E. coli* ATCC 25922, *Pseudomonas aeruginosa* ATCC 27853, *Klebsiella pneumoniae* ATCC 13883, *Proteus mirabilis* ATCC 29906, *Acinetobacter baumannii* ATCC 19606, *Salmonella enterica* ATCC 25928, and *Salmonella* typhimurium ATCC 14028. The yeast panel consisted of *Candida albicans* ATCC 10231 and *Candida parapsilosis* ATCC 22019.

### 2.7. Antimicrobial Activity

The antimicrobial activity of 10% propolis extracts and extract combinations prepared as described in Section 2.2 was evaluated against the microbial strains mentioned in 2.6. The pathogenic strains were aerobically cultured in 10 mL of BHI broth at 37 °C for 16 h. Bacterial suspensions were adjusted to a concentration of 0.5 McFarland to give a cell density of ~10^8^ CFU/mL. The antimicrobial activity of propolis samples was performed according to Iorizzo et al. [31] with some modifications. Briefly, 15 mL of BHI agar was inoculated with a 0.5 McFarland of pathogens to Petri dishes (Ø 90 mm). Wells of 6 mm diameter were cut in the plates, and 50 μL of propolis extract was added to the wells. As a negative control, 50 μL of 80% ethanol was used, while as a positive control, vancomycin (30 μg) was used for Gram-positive bacteria, gentamicin (10 μg) for Gram-negative bacteria, and fluconazole (25 μg) for yeasts.

Following incubation at 37 °C for 24–48 h, antimicrobial activity was evaluated by measuring the diameter of the clear zone of inhibition (ZOI) surrounding each well, expressed in mm. The total diameter of the inhibition area was measured in mm using a digital caliper. The diameter of the well was subsequently subtracted from the measured value, and the resulting value was reported as ZOI. All assays were performed in triplicate.

### 2.8. Minimum Inhibitory Concentration

The minimum inhibitory concentration (MIC) of the propolis extracts was determined using a broth microdilution assay in 96-well microtiter plates. The procedure was based on the Clinical and Laboratory Standards Institute (CLSI) guidelines M07-A9 and M27-A3 [32,33] with modifications as described by Torres et al. [34].

Bacterial and fungal strains were cultured in Brain Heart Infusion (BHI) broth and Sabouraud Dextrose Broth (SDB), respectively. Propolis extracts were tested at final concentrations ranging from 50 mg/mL to 0.01 mg/mL. The assays were performed in sterile 96-well microplates and incubated at 37 °C for 24 h. Bacterial inocula were standardised to approximately1 × 10^8^ CFU/mL using the 0.5 McFarland standard and subsequently diluted to achieve a final concentration of 5 × 10^5^ CFU/mL in each well. Fungal inocula were standardised to approximately 1 × 10^6^ CFU/mL using the same procedure. The propolis extracts were prepared in 80% ethanol, and the concentrations reported above corresponded to the final propolis concentrations in the wells.

Following incubation, 30 μL of 0.01% (*w*/*v*) resazurin solution was added to each well, and the plates were incubated for a further 30 min. Microbial growth was assessed visually according to the colour of the resazurin indicator. A colour change from purple to pink was interpreted as evidence of microbial metabolic activity, and consequently, microbial growth. The MIC was defined as the lowest concentration of propolis extract at which no visible evidence of microbial growth was observed.

For quality control, each assay included a growth control containing the microbial inoculum without propolis extract, a sterility control containing culture medium without microorganisms, and an ethanol vehicle control corresponding to the solvent used for extraction and dilution of the propolis samples. The growth control was used to verify microbial growth under the assay conditions, whereas the sterility control was used to verify the absence of contamination in the culture medium. The ethanol vehicle control was included to determine whether the extraction/dilution solvent itself contributed to the observed antimicrobial effect. Reference antimicrobial agents were also included: vancomycin for Gram-positive bacteria, gentamicin for Gram-negative bacteria, and fluconazole for yeasts.

MIC determinations for each treatment–microorganism combination were performed in three independent experiments.

### 2.9. Fractional Inhibitory Concentration Index for Predefined Ratios

The interactions between individual propolis extracts and their defined mixtures were evaluated using the fractional inhibitory concentration index (FICI) approach. FICI values were calculated separately for each extract combination and each tested microorganism using MIC values for the predefined 1:1 fixed-ratio combination assay for binary combinations and a predefined 1:1:1:1:1 ratio for the PAN mix.

For the binary combinations, the fractional inhibitory concentration (FIC) of each constituent extract was calculated as the ratio between the concentration of that extract present in the combination at the inhibitory endpoint and its MIC when tested individually:

FIC_A_ = MIC_A_ (A, combination)/MIC_A_ (A, alone)

FIC_B_ = MIC_B_ (B, combination)/MIC_B_ (B, alone)

The FICI for each binary combination was calculated as

FICI = FIC_A_ + FIC_B_

For PAN, consisting of the five propolis extracts combined in equal proportions (1:1:1:1:1), the FIC of each constituent extract was calculated as the ratio between its concentration in the PAN mixture at the inhibitory endpoint and its MIC when tested individually. The FICI for PAN was therefore calculated as the sum of the FICs of its five constituent extracts:

$${\mathrm{FICI}}_{\mathrm{PAN}}=\sum_{i=1}^{5}\frac{{\mathrm{MIC}}_{\mathrm{PAN}}/5}{{\mathrm{MIC}}_{i}}$$

FICI values were calculated independently for each microorganism and each tested combination. The resulting values were classified according to the predefined interpretative criteria—synergy (FICI ≤ 0.50), additive effect (0.5 < FICI ≤ 1.00), indifference (1.00 < FICI ≤ 4.00), and antagonism (FICI > 4.00)—based on previously reported classification schemes [32,33], and the results are restricted to the predefined combinations and ratios performed in this study.

### 2.10. Statistical Analysis

Statistical analyses were performed in R version 4.6.1 (R Core Team, 2026). TPC, TFC, DPPH, ABTS, FRAP, and ORAC values were analysed separately using one-way analysis of variance (ANOVA), with sample or experimental group as the fixed factor. Model assumptions were assessed using the Shapiro–Wilk test for residual normality and Levene’s test for homogeneity of variances; Bartlett’s test was additionally applied to the ORAC data. When significant differences were detected, Tukey’s honestly significant difference (HSD) test was used for pairwise comparisons. Results are presented as mean ± standard deviation (SD). Statistical significance was set at *p* < 0.05.

For the HPLC dataset, differences in the concentrations of the 20 quantified polyphenolic compounds among the 16 propolis preparations were assessed using the Kruskal–Wallis test. Significant Kruskal–Wallis tests were followed by Dunn’s multiple-comparison test with Holm adjustment. To account for multiple testing across the 20 compounds, omnibus *p*-values were additionally adjusted using the Benjamini–Hochberg false discovery rate (FDR) procedure. Compact-letter displays were generated based on the Holm-adjusted pairwise comparisons. Statistical significance was defined as an FDR-adjusted *p* < 0.05.

Multivariate analyses were performed separately for the chemical and biological datasets using principal component analysis (PCA) in R with the FactoMineR package. The chemical dataset comprised 26 variables, including TPC, TFC, DPPH, ABTS, FRAP, ORAC, and 20 HPLC-quantified polyphenolic compounds. Chemical variables were centred and scaled to unit variance before PCA. The biological dataset comprised the antimicrobial response variables and was similarly centred and scaled. Spearman rank correlations were calculated between the scores of the first three chemical and biological principal components. The resulting *p*-values were adjusted for multiple testing using the Benjamini–Hochberg FDR procedure, with an FDR-adjusted *p* < 0.05 considered statistically significant. Partial least-squares (PLS) regression was subsequently performed separately for each biological response, using the chemical variables as predictors. Models were fitted using up to three latent components with cross-validation, and variable importance in projection (VIP) scores were calculated to assess the relative contribution of individual chemical variables to each biological response. A VIP > 1 was considered indicative of relatively high variable importance.

## 3. Results

### 3.1. Total Phenolic Content and Total Flavonoid Content

Significant differences among the propolis extracts and their combinations were observed for both total phenolic content (TPC) and total flavonoid content (TFC) (Table 1; *p* < 0.05).

The TPC ranged from 88.8 ± 0.3 mg GAE/g in C2 to 149.8 ± 0.5 mg GAE/g in A. Among the individual extracts, A showed the highest TPC, followed by H (147.8 ± 0.8 mg GAE/g), C1 (126.5 ± 0.3 mg GAE/g), and S (124.8 ± 0.5 mg GAE/g). Among the combinations, A + H and S + H showed relatively high TPC values (141.9 ± 0.5 and 143.2 ± 0.5 mg GAE/g, respectively), whereas C2 + S had the lowest value among the combinations (93.8 ± 0.7 mg GAE/g).

The TFC showed a different distribution pattern. The highest values were recorded for C1 (20.0 ± 0.4 mg QE/g) and C2 (19.9 ± 0.1 mg QE/g), which did not differ significantly from each other. In contrast, H showed the lowest TFC (2.4 ± 0.4 mg QE/g), followed by S (5.4 ± 0.2 mg QE/g). The binary combinations exhibited intermediate values, ranging from 4.0 ± 0.2 mg QE/g for S + H to 15.6 ± 0.8 mg QE/g for C1 + A. Overall, the Tukey groupings demonstrated distinct patterns of variation between TPC and TFC across the individual extracts and their combinations.

### 3.2. Antioxidant Activity

Antioxidant activity differed significantly among the propolis extracts and their combinations, although the relative ranking of treatments varied among the assays (Table 2).

For DPPH, the highest values were recorded for A + H (30.5 ± 0.2), C1 + H (30.3 ± 0.5), C2 + A (30.0 ± 0.4), A (29.7 ± 0.1), and A + S (29.6 ± 0.4), which largely shared overlapping Tukey groupings. In contrast, S showed the lowest DPPH value (7.9 ± 0.5), followed by C2 (19.0 ± 0.6). Several significant differences were observed among the treatments, as indicated by the distinct compact-letter groupings.

The ABTS values ranged from 28.4 ± 0.6 in C2 to 36.5 ± 0.3 in A. Samples C1 and A + S also showed relatively high values (35.9 ± 0.3 and 35.9 ± 0.7, respectively), whereas C2 + S (28.5 ± 0.8), C1 + S (28.8 ± 0.5), and C2 (28.4 ± 0.6) were among the lowest. The Tukey analysis indicated significant differences among several treatments, while some groups with similar values did not differ significantly.

For FRAP, the values ranged from 4.6 ± 0.3 in C2 + S to 7.6 ± 0.3 in A. Sample S also showed a high FRAP value (7.2 ± 0.5) and shared a Tukey grouping with A, indicating no significant difference between these two extracts. H (6.5 ± 0.4), A + H (6.6 ± 0.3), and C1 (6.4 ± 0.6) showed intermediate reducing capacities, whereas C2 + S, C1 + S, and C2 + H were among the treatments with lower values.

The ORAC values showed a pronounced separation among experimental groups. One-way ANOVA revealed a highly significant effect of experimental group on ORAC values (F_15,48_ = 1443.79, *p* < 0.0001), with a very large effect size (η^2^ = 0.998).

The highest ORAC value was observed for A + S (3213.74 ± 11.47 mg TE/g), which differed significantly from all other groups according to Tukey’s HSD test. Among the individual extracts, H showed the highest ORAC value (2970.77 ± 21.61 mg TE/g), followed by C1 (2906.64 ± 12.76 mg TE/g) and S (2881.06 ± 19.04 mg TE/g). Sample H differed significantly from both C1 and S, whereas C1 and S were not significantly different. The lowest ORAC values were observed for C2 (2194.51 ± 16.70 mg TE/g) and C1 + C2 (2206.01 ± 16.71 mg TE/g). Among the binary combinations, A + S showed the highest ORAC value, whereas C1 + C2 and C2 + S showed comparatively lower values.

Overall, the four antioxidant assays produced distinct treatment rankings, with significant differences observed among the extracts and combinations for each assay.

### 3.3. Phenolic Profile of the PPEs

**Figure 1:**
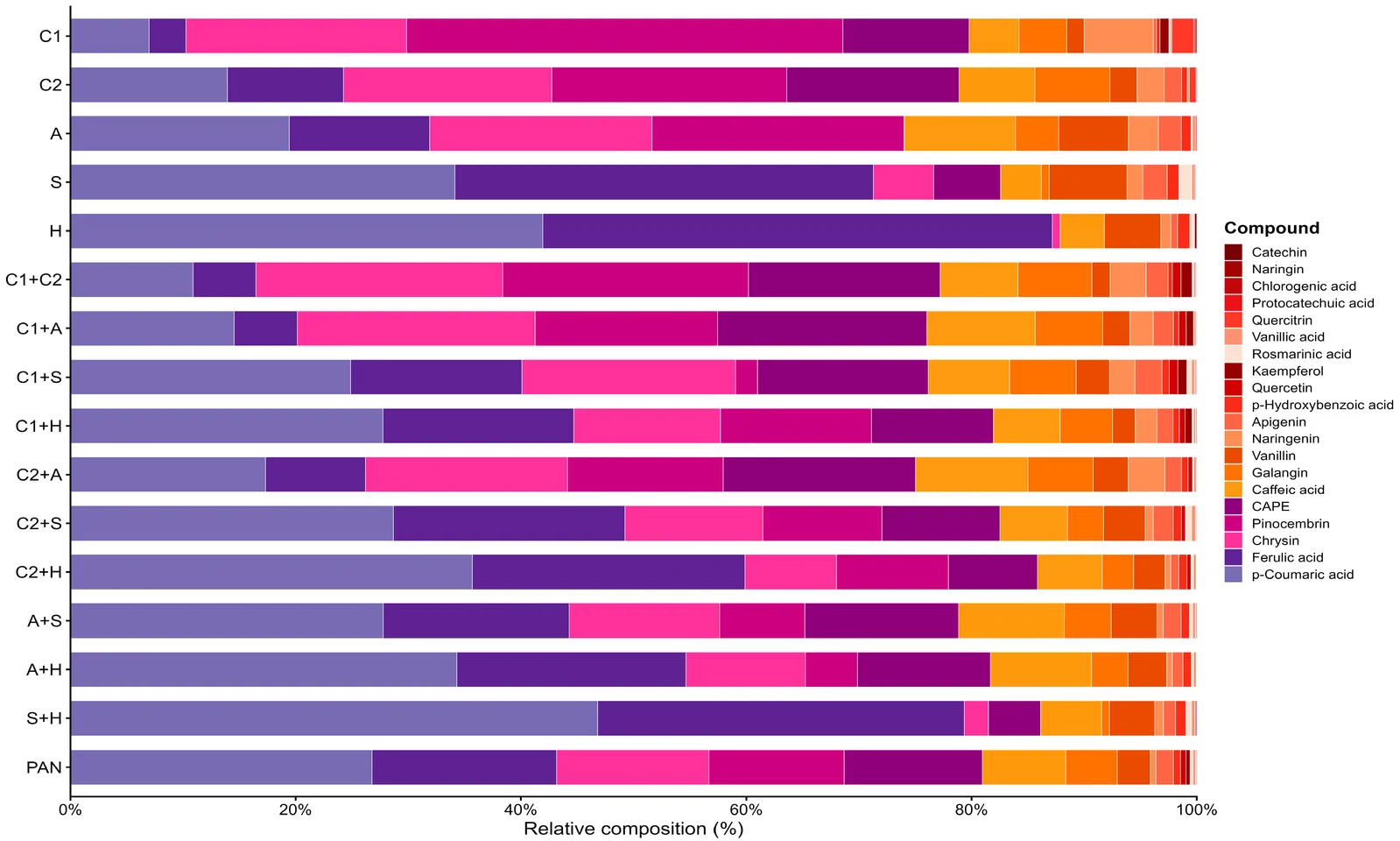
Phenolic profiles of the five individual propolis ethanolic extracts and mixes. The data were expressed as the mean ± SD (*n* = 3).

The HPLC analysis revealed significant differences in the concentrations of individual polyphenolic compounds among the propolis extracts and their combinations (Supplementary Table S1; Figure 1). The magnitude and direction of these differences varied considerably among compounds.

Among the flavonoids, apigenin concentrations ranged from 2.73 µg/g in C1 to 32.10 µg/g in C1 + A, with C1 + A showing a significantly higher concentration than C1. CAPE reached its highest detected concentration in C1 + C2 (332.73 µg/g). However, significant differences were mainly observed between C1 + C2 and A. Chrysin showed a similar range of variation, with the highest concentration recorded in C1 + A (377.79 µg/g), significantly exceeding that detected in H (5.38 µg/g). Galangin concentrations also differed significantly among formulations, ranging from 4.19 µg/g in S to 107.15 µg/g in C1 + C2. Naringenin was highest in C1 (62.11 µg/g), with significantly higher concentrations than those observed in H (6.92 µg/g) and C2 + H (6.67 µg/g). Pinocembrin showed substantial variation, ranging from 25.25 µg/g in C1 + A to 391.44 µg/g in C1, with a significant difference between these formulations.

Among the phenolic acids, caffeic acid reached its highest concentration in C1 + A (172.18 µg/g), significantly exceeding the concentrations detected in S and H. Ferulic acid was highest in S + H (437.29 µg/g), while the lowest detected concentration was observed in C1 (33.09 µg/g). p-Coumaric acid showed particularly high concentrations in S + H (629.29 µg/g) and A + H (545.14 µg/g), compared with 70.60 µg/g in C1. Vanillic acid and vanillin also differed significantly among formulations, with the highest concentrations detected in A + H (4.10 and 54.69 µg/g, respectively).

Several compounds showed restricted detection across the tested formulations. Catechin, kaempferol, naringin, quercitrin, and quercetin were detected only in subsets of the samples. Quercitrin, for example, was detected in C1 and C2, with a higher concentration in C1 than in C2 (19.70 and 6.09 µg/g, respectively).

Overall, the HPLC profiles showed substantial compound-specific variation among the individual extracts and their combinations. Although some combinations exhibited higher concentrations of specific polyphenols than individual extracts, these differences were not consistent across compounds, and statistical significance was observed only for selected pairwise comparisons.

### 3.4. Antimicrobial Activity of Propolis Extracts

**Figure 2:**
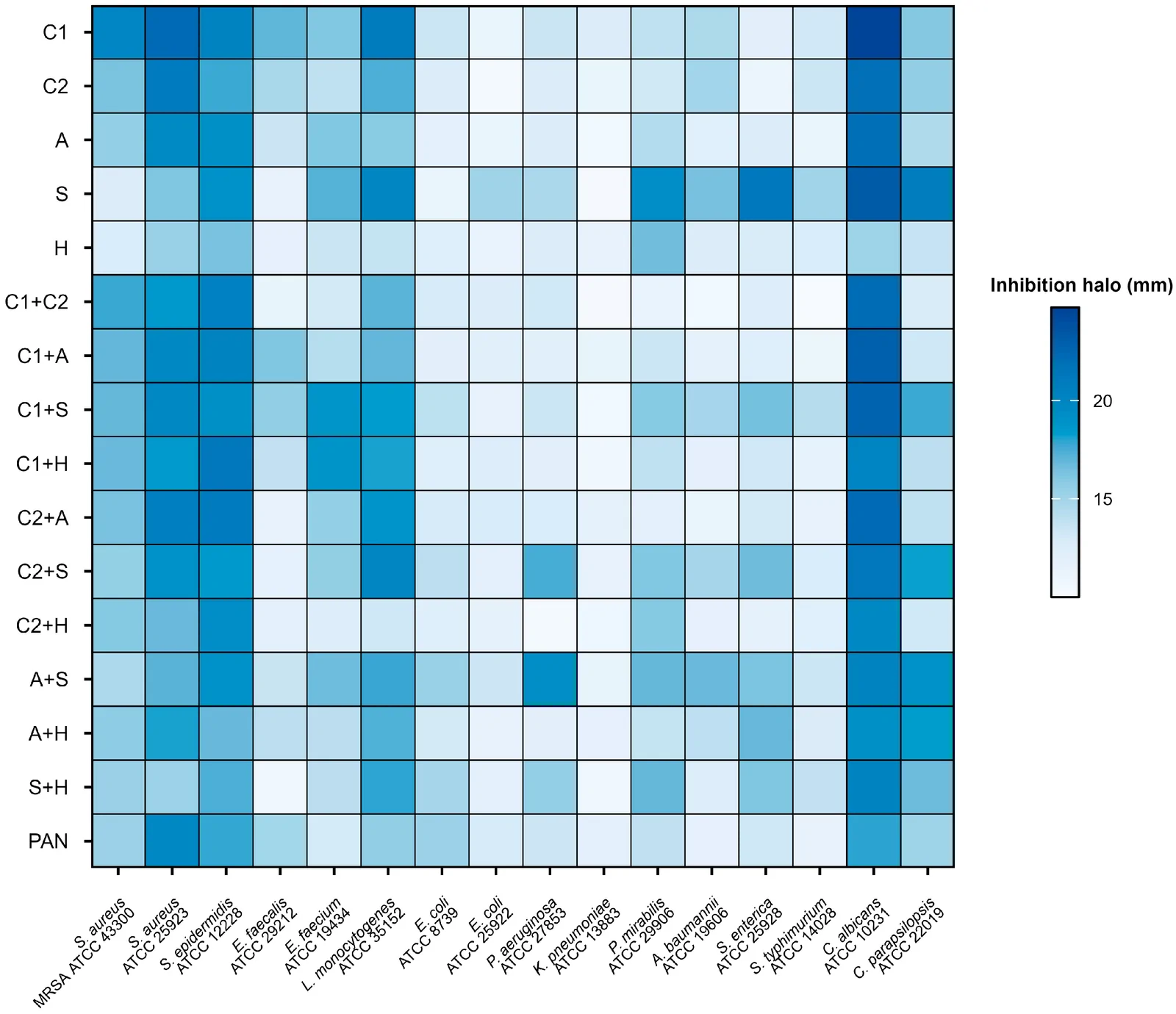
Heatmap showing the inhibition zones (mm) produced by individual propolis extracts and their mixtures against Gram-positive and Gram-negative pathogenic bacteria and yeast reference strains.

The inhibition-zone assay showed substantial variation among treatments and microorganisms (Supplementary Tables S2 and S3; Figure 2). Significant differences among PEEs were observed for most microorganisms. The magnitude of inhibition varied markedly across microorganisms, with distinct activity patterns among individual extracts and their combinations.

Among the individual PEEs, C1 generally produced comparatively large inhibition zones, particularly against *C. albicans* ATCC 10231 (24.73 ± 0.41 mm), *S. aureus* ATCC 25923 (22.21 ± 0.50 mm), *L. monocytogenes* ATCC 35152 (21.09 ± 0.01 mm), and *S. epidermidis* ATCC 12228 (20.32 ± 0.44 mm). Extract C2 also showed pronounced inhibition against *S. aureus* ATCC 25923 (20.93 ± 0.08 mm) and *C. albicans* (22.00 ± 0.05 mm) but generally produced smaller inhibition zones against several Gram-negative bacteria. Extract A showed relatively high activity against *C. albicans* (22.07 ± 0.10 mm), *S. aureus* ATCC 25923 (19.62 ± 0.26 mm), and *S. epidermidis* ATCC 12228 (19.30 ± 0.25 mm). In contrast, S exhibited comparatively high inhibition against several Gram-negative microorganisms, including *S. enterica* ATCC 25928 (21.15 ± 0.08 mm) and *P. mirabilis* ATCC 29906 (19.36 ± 0.35 mm), as well as *C. parapsilosis* ATCC 22019 (20.75 ± 0.43 mm). Extract H generally showed lower inhibition zones across several tested microorganisms. The binary combinations also displayed microorganism-dependent activity profiles. Mix C1 + S showed relatively high inhibition against *S. aureus* ATCC 25923 (19.80 ± 0.41 mm), *E. faecium* ATCC 19434 (18.76 ± 0.08 mm), and *L. monocytogenes* ATCC 35152 (18.35 ± 0.34 mm). Mix C2 + S produced notable inhibition against *P. aeruginosa* ATCC 27853 (17.57 ± 0.36 mm), *S. enterica* ATCC 25928 (16.73 ± 0.25 mm), and *P. mirabilis* ATCC 29906 (16.05 ± 0.09 mm), while also showing inhibition zones of 19.05 ± 0.28 mm against *S. aureus* ATCC 25923 and 19.99 ± 0.38 mm against *L. monocytogenes*. Among the A-containing combinations, A + S showed comparatively high activity against *P. aeruginosa* (19.41 ± 0.24 mm), *P. mirabilis* (16.88 ± 0.18 mm), *A. baumannii* (16.80 ± 0.30 mm), and *S. enterica* (16.36 ± 0.19 mm). It also produced inhibition zones of 20.18 ± 0.68 mm and 19.02 ± 0.16 mm against *C. albicans* and *C. parapsilosis*, respectively.

Across the microbial groups, Gram-positive bacteria generally exhibited larger inhibition zones than several Gram-negative strains, particularly for C1. The highest inhibition among the Gram-positive microorganisms was observed for C1 against *S. aureus* ATCC 25923 (22.21 ± 0.50 mm), followed by *L. monocytogenes* (21.09 ± 0.01 mm). Responses among Gram-negative bacteria were more heterogeneous and depended strongly on the microorganism and treatment. Extract S showed comparatively high activity against *S. enterica* and *P. mirabilis*, whereas A + S produced the largest inhibition among the combinations against *P. aeruginosa*. The two yeast species also exhibited distinct susceptibility patterns. For *C. albicans*, C1 produced the largest inhibition zone (24.73 ± 0.41 mm), followed by C1 + S (22.73 ± 0.23 mm), A (22.07 ± 0.10 mm), and C2 (22.00 ± 0.05 mm). For *C. parapsilosis*, the highest inhibition was observed for S (20.75 ± 0.43 mm), followed by A + S (19.02 ± 0.16 mm) and C2 + S (18.20 ± 0.09 mm).

Overall, the inhibition-zone data demonstrated pronounced treatment- and microorganism-dependent variation. Extract C1 showed comparatively high inhibition against several Gram-positive bacteria and *C. albicans*, whereas S and selected S-containing combinations showed comparatively high activity against several Gram-negative microorganisms. The combination treatments produced distinct inhibition profiles across the microbial panel, with no uniform increase in inhibition relative to the corresponding individual extracts.

### 3.5. Minimum Inhibitory Concentration

**Figure 3:**
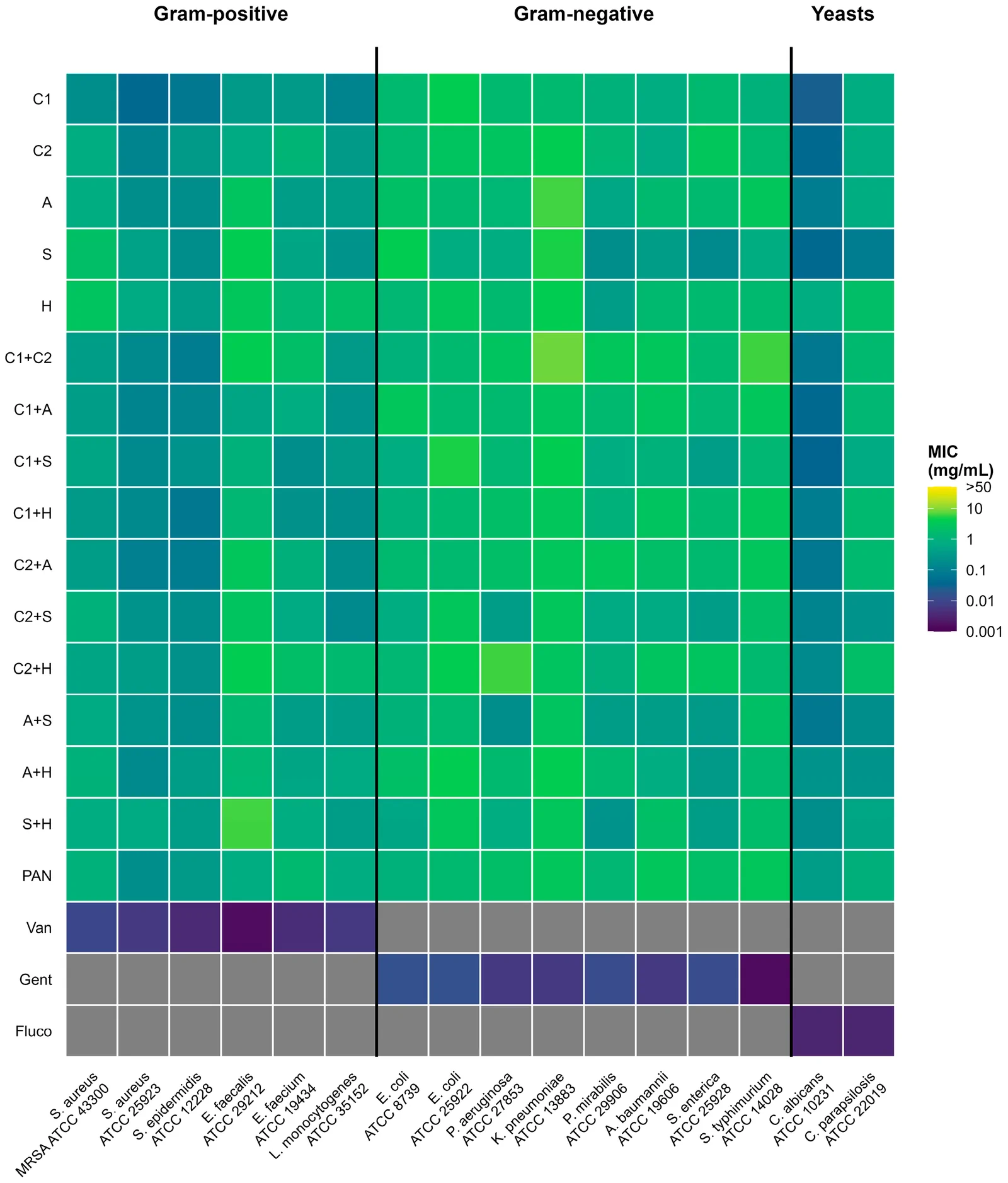
Heatmap of log_2_-transformed MIC values for individual propolis extracts, binary combinations, PAN, and reference controls against the tested microorganisms. Lower log_2_(MIC) values indicate lower MICs and therefore greater inhibitory activity.

The antimicrobial activity of the propolis extracts and their combinations was evaluated against 16 reference microorganisms by determining the minimum inhibitory concentration (MIC) (Supplementary Tables S4 and S5; Figure 3). The MIC values varied substantially among both treatments and microorganisms, indicating distinct susceptibility patterns across the tested microbial panel.

Among Gram-positive bacteria, the lowest MIC values were generally observed for *S. aureus* ATCC 25923 and *S. epidermidis* ATCC 12228. C1 showed particularly low MICs against these strains (0.04 and 0.0567 mg/mL, respectively) and also showed strong activity against MRSA (*S. aureus* ATCC 43300; 0.19 mg/mL) and *L. monocytogenes* ATCC 35152 (0.16 mg/mL). C1-containing combinations also showed low MICs against several Gram-positive strains, including C1 + A against *S. aureus* ATCC 25923 (0.09 mg/mL). Activity against enterococci was more variable, with MIC values ranging from 0.39 to 6.25 mg/mL across treatments.

Gram-negative bacteria showed greater variability in susceptibility. *P. aeruginosa* ATCC 27853 showed low MICs with A + S (0.19 mg/mL) and C2 + S (0.39 mg/mL), while *P. mirabilis* ATCC 29906 showed low MICs with S (0.19 mg/mL), A + S (0.39 mg/mL), and S + H (0.26 mg/mL). In contrast, *K. pneumoniae* ATCC 13883 and *E. coli* ATCC 25922 generally showed higher MIC values. The highest MIC among the propolis treatments was observed for *K. pneumoniae* with C1 + C2 (8.33 mg/mL). For *A. baumannii* ATCC 19606, the lowest MIC values were observed with S and A + S (0.39 mg/mL) and A + H (0.78 mg/mL). Yeasts generally showed low MIC values across several treatments. *Candida albicans* ATCC 10231 was particularly susceptible to C1 (0.0267 mg/mL), C2 (0.04 mg/mL), C1 + A (0.04 mg/mL), and C1 + S (0.0333 mg/mL). Low MICs were also observed with C2 + A and A + S (0.0733 mg/mL). *C. parapsilosis* ATCC 22019 generally showed higher MIC values than *C. albicans*, although low MICs were observed for S (0.09 mg/mL), A + S (0.19 mg/mL), C2 + S (0.26 mg/mL), and A + H (0.26 mg/mL).

Overall, C1 showed a comparatively strong activity profile, particularly against several Gram-positive bacteria and *C. albicans*. The activity of the other individual extracts and binary combinations was more heterogeneous and strongly dependent on the microorganism. Notably, combinations did not consistently produce lower MIC values than the corresponding individual extracts. The observed MIC patterns therefore indicate treatment- and microorganism-dependent differences in susceptibility, while the nature of interactions among extracts is addressed separately through FICI analysis.

### 3.6. Multivariate Analysis of Chemical and Biological Profiles

**Figure 4:**
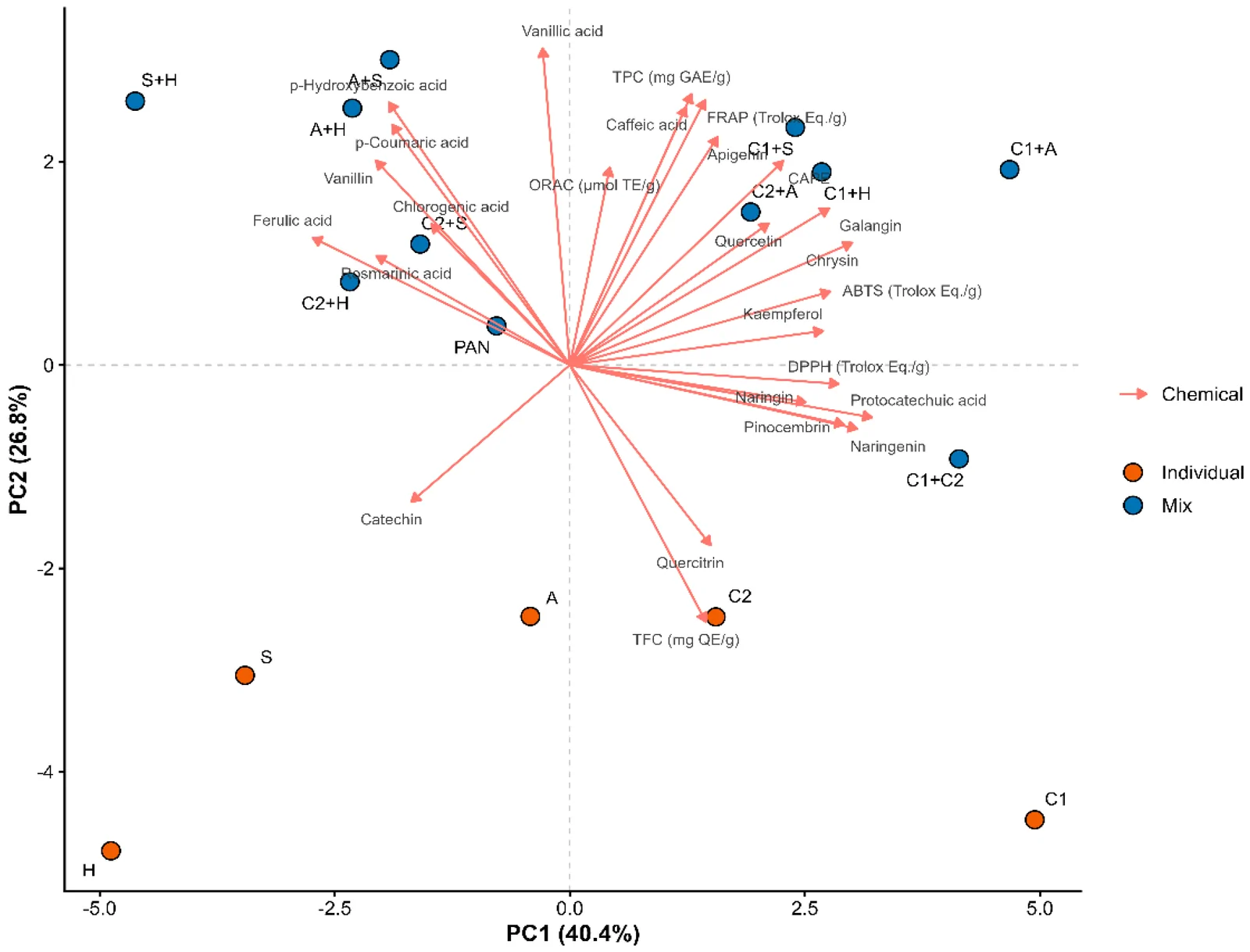
Principal component analysis (PCA) of the chemical profiles of the propolis extracts and combinations.

Principal component analysis (PCA) revealed distinct multivariate patterns in the chemical and biological datasets. For the chemical dataset, PC1 and PC2 explained 40.4% and 26.8% of the total variance, respectively, accounting for 67.2% cumulatively (Figure 4).

The chemical score plot showed clear differentiation among individual extracts and mixtures, with C1 positioned at the positive extreme of PC1, whereas S and H were located at negative PC1 scores. Most C1-containing mixtures also showed positive PC1 scores, while several combinations involving C2, A, S, and H occupied the negative PC1 region. PC2 further contributed to the discrimination of the samples, with the individual extracts generally located at negative PC2 scores and most mixtures shifted towards positive PC2 values. The corresponding loading structure showed that PC1 was mainly associated with protocatechuic acid, naringenin, pinocembrin, chrysin, galangin, kaempferol, DPPH, and ABTS, whereas PC2 was primarily associated with TPC, TFC, FRAP, vanillic acid, p-hydroxybenzoic acid, caffeic acid, p-coumaric acid, and apigenin.

**Figure 5:**
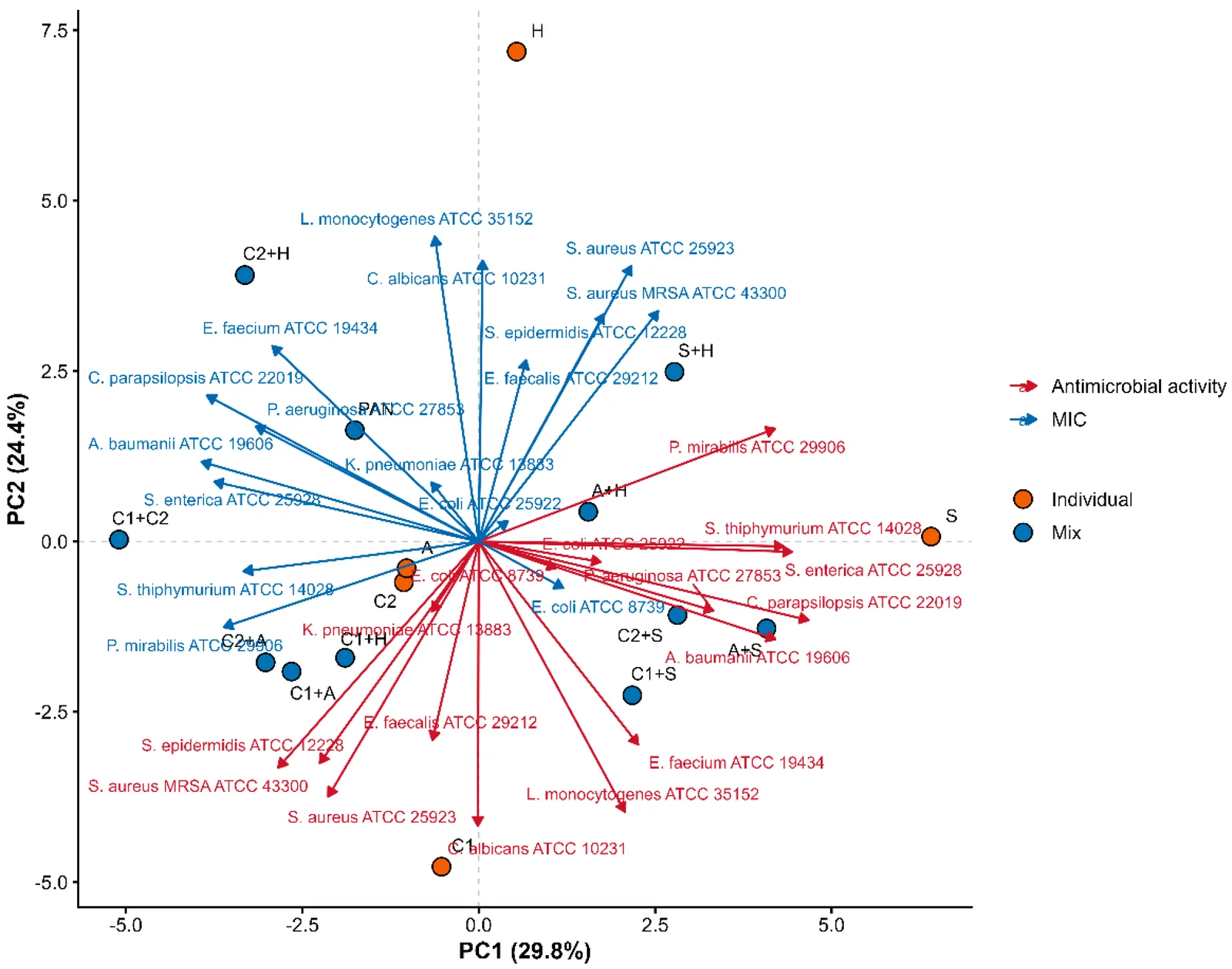
Principal component analysis (PCA) of the biological profiles of the propolis extracts and combinations.

The biological PCA showed a distinct multivariate organisation, with PC1 and PC2 explaining 29.80% and 24.35% of the total variance, respectively (54.15% cumulatively) (Figure 5).

Individual extracts and mixtures were distributed across the two-dimensional space according to their composite antimicrobial-response profiles, with S showing a pronounced positive PC1 score and H characterised by a high positive PC2 score. In contrast, several mixtures, including C1 + C2, C1 + A, and C2 + A, showed negative PC1 scores. The biological loading pattern indicated contributions from inhibition-zone and MIC responses across Gram-positive bacteria, Gram-negative bacteria, and yeasts.

**Figure 6:**
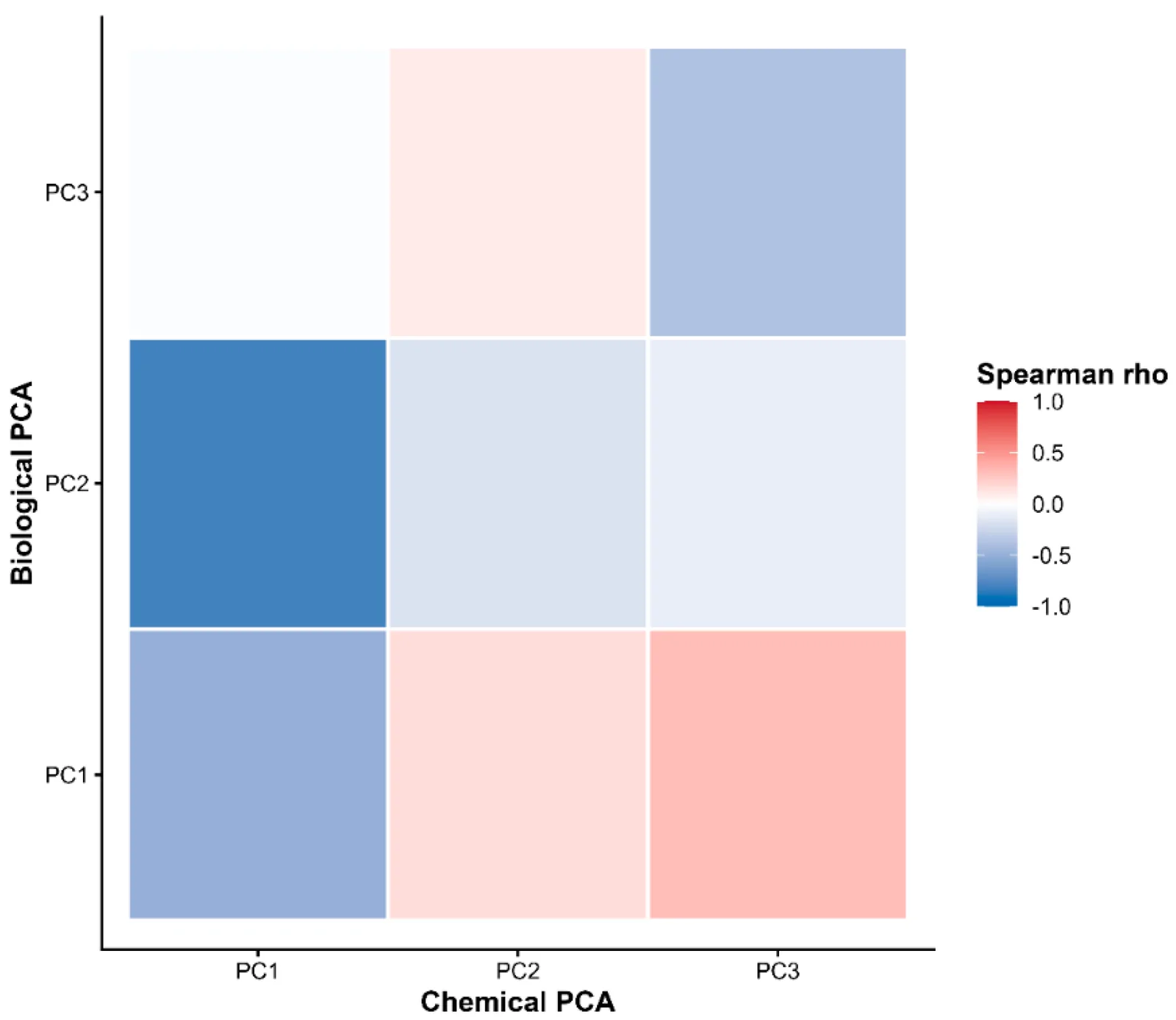
Correlation between the chemical and biological PCA spaces. Spearman rank correlations between the principal component scores obtained from the chemical and biological datasets.

To directly evaluate the relationship between the two multivariate datasets, Spearman correlations were calculated between the corresponding PCA scores (Figure 6). A strong inverse association was observed between chemical PC1 and biological PC2 (ρ = −0.809, adjusted *p* = 0.0013), whereas none of the other PC1–PC2 associations remained significant after adjustment for multiple comparisons.

**Figure 7:**
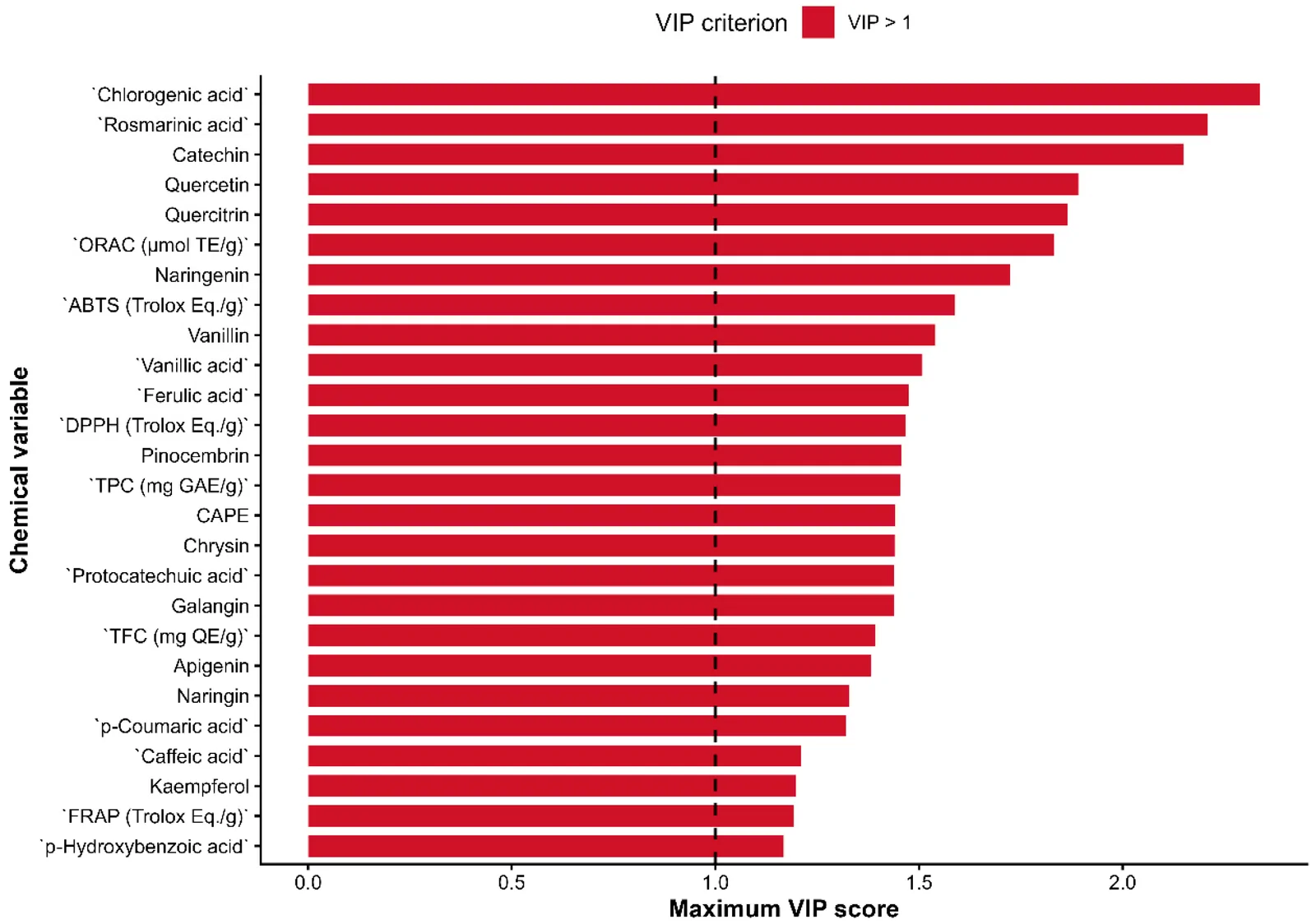
Partial least squares (PLS) analysis linking chemical variables to biological-response profiles. Variable importance in projection (VIP) scores obtained from the exploratory PLS model. Variables with mean VIP values >1 are shown, indicating above-average contribution to the fitted model.

An exploratory partial least squares (PLS) analysis was subsequently performed to identify chemical variables contributing to variation in the biological-response dataset. Variables with VIP > 1 included rosmarinic acid (mean VIP = 1.183), ABTS (1.129), catechin (1.117), pinocembrin (1.105), chlorogenic acid (1.072), naringenin (1.065), DPPH (1.052), quercetin (1.038), ferulic acid (1.011), protocatechuic acid (1.003), and chrysin (1.000). Among these variables, the highest individual VIP values were observed for chlorogenic acid (VIP = 2.337), rosmarinic acid (VIP = 2.209), catechin (VIP = 2.149), and quercetin (VIP = 1.892) for selected microorganism-specific biological responses (Figure 7). The complete three-dimensional PCA plots and detailed PLS-VIP profiles are reported in the Supplementary Materials (Figures S6–S12).

### 3.7. Fractional Inhibitory Concentration Index Results for Predefined Ratios

For all the mixes, FICI values were obtained and interpreted according to the criteria described in Section 2.9. Overall, for the predefined ratios tested, the analysis revealed predominant indifference to additive interactions across the PPE combinations against the tested microbial strains (Supplementary Tables S13 and S14). Indifferent interactions represented the most frequent outcome, accounting for 60.8% of all tests, indicating that the majority of combinations maintained their antimicrobial activity without evidence of antagonistic interference. Additive interaction accounted for 25.0% of the observed interactions, while 9.09% were classified as synergistic. Together, these two types of interaction represented more than one third of the tested combinations, whereas antagonistic interactions accounted for only 5.11%.

Overall, the results indicate good functional compatibility among the combinations tested. The majority of PPE interactions were indifferent, indicating maintenance of antimicrobial activity, while one-fourth of the combinations showed either an additive effect or enhanced antimicrobial activity beyond the individual effects, corresponding to additive and synergistic interactions, respectively. The low proportion of antagonistic interactions suggests good compatibility when different extracts are combined, with a relatively small proportion of mixtures resulting in reduced antimicrobial activity.

Of the 9 antagonistic interactions, four were observed for the C1 + C2 combination against the following strains: *E. faecalis* ATCC 29212 (FICI = 8.533), *S. typhimurium* ATCC 14028 (FICI = 6.010), *E. faecium* ATCC 19434 (FICI = 4.889) and *A. baumannii* ATCC 19606 (FICI = 4.800). Two antagonistic interactions were observed for C1 + S against *C. parapsilosis* ATCC 22019 (FICI = 4.028) and *E. coli* ATCC 25922 (FICI = 4.342). For PAN, two antagonistic interactions were found for *C. albicans* ATCC 10231 (FICI = 7.788) and *A. baumannii* ATCC 19606 (FICI = 4.320). One antagonistic interaction was observed with the C2 + A mix against *P. mirabilis* ATCC 29906 (FICI = 4.200).

A total of 16 synergistic interactions were observed. Four occurred for the A + S combination against *E. faecalis* ATCC 29212 (FICI = 0.488), *E. coli* ATCC 8739 (FICI = 0.375), *P. aeruginosa* ATCC 27853 (FICI = 0.195), and *K. pneumoniae* ATCC 13883 (FICI = 0.458). Four synergistic interactions were observed for A + H against *E. faecalis* ATCC 29212 (FICI = 0.458), *A. baumannii* ATCC 19606 (FICI = 0.500), *S. enterica* ATCC 25928 (FICI = 0.500), and *C. parapsilosis* ATCC 22019 (FICI = 0.229). For S + H, two synergistic interactions were observed against *S. aureus* MRSA ATCC 43300 (FICI = 0.338) and *E. coli* ATCC 8739 (FICI = 0.263). Two synergistic interactions were observed for C2 + S against *E. coli* ATCC 8739 (FICI = 0.344) and *P. aeruginosa* ATCC 27853 (FICI = 0.325). For C2 + A, two synergistic interactions were observed against *S. aureus* MRSA ATCC 43300 (FICI = 0.500) and *S. epidermidis* ATCC 12228 (FICI = 0.377). The remaining synergistic interactions were observed for C1 + S against *E. coli* ATCC 8739 (FICI = 0.344) and for C2 + H against *S. aureus* MRSA ATCC 43300 (FICI = 0.433). The strongest synergistic interaction was observed for A + S against *P. aeruginosa* ATCC 27853 (FICI = 0.195), followed by A + H against *C. parapsilosis* ATCC 22019 (FICI = 0.229) and S + H against *E. coli* ATCC 8739 (FICI = 0.263).

As far as additivity is concerned, a total of 44 interactions were observed across the mixtures. For C1 + C2, additive interactions were observed against *S. epidermidis* ATCC 12228 (FICI = 0.934), *E. coli* ATCC 8739 (FICI = 0.667), *E. coli* ATCC 25922 (FICI = 0.600), and *S. enterica* ATCC 25928 (FICI = 0.750). For C1 + A, additive interactions were observed against *E. faecalis* ATCC 29212 (FICI = 0.767), *C. albicans* ATCC 10231 (FICI = 0.971), *E. coli* ATCC 25922 (FICI = 0.800), and *S. enterica* ATCC 25928 (FICI = 0.833). For C1 + S, an additive interaction was observed against *E. faecium* ATCC 19434 (FICI = 0.548). For C1 + H, additive interactions were observed against *S. epidermidis* ATCC 12228 (FICI = 0.740), *E. faecium* ATCC 19434 (FICI = 0.508), *L. monocytogenes* ATCC 35152 (FICI = 0.639), *E. coli* ATCC 8739 (FICI = 0.733), and *E. coli* ATCC 25922 (FICI = 0.550). For C2 + A, additive interactions were observed against *S. aureus* ATCC 25923 (FICI = 0.748), *L. monocytogenes* ATCC 35152 (FICI = 0.540), *E. coli* ATCC 8739 (FICI = 0.875), *E. coli* ATCC 25922 (FICI = 0.800), *K. pneumoniae* ATCC 13883 (FICI = 0.625), and *S. enterica* ATCC 25928 (FICI = 0.750). For C2 + S, additive interactions were observed against *S. aureus* MRSA ATCC 43300 (FICI = 0.917), *S. epidermidis* ATCC 12228 (FICI = 0.797), *E. faecium* ATCC 19434 (FICI = 0.903), *L. monocytogenes* ATCC 35152 (FICI = 0.558), and *K. pneumoniae* ATCC 13883 (FICI = 0.674). For C2 + H, additive interactions were observed against *S. epidermidis* ATCC 12228 (FICI = 0.740), *E. coli* ATCC 8739 (FICI = 0.917), and *K. pneumoniae* ATCC 13883 (FICI = 0.625).

For A + S, additive interactions were observed against *S. aureus* MRSA ATCC 43300 (FICI = 0.573), *S. aureus* ATCC 25923 (FICI = 0.973), *E. faecium* ATCC 19434 (FICI = 0.875), and *A. baumannii* ATCC 19606 (FICI = 0.625). For A + H, additive interactions were observed against *S. aureus* MRSA ATCC 43300 (FICI = 0.867), *S. aureus* ATCC 25923 (FICI = 0.544), *L. monocytogenes* ATCC 35152 (FICI = 0.990), *K. pneumoniae* ATCC 13883 (FICI = 0.833), and *S. typhimurium* ATCC 14028 (FICI = 0.850). For S + H, additive interactions were observed against *L. monocytogenes* ATCC 35152 (FICI = 0.844), *P. aeruginosa* ATCC 27853 (FICI = 0.750), and *K. pneumoniae* ATCC 13883 (FICI = 0.674). Finally, PAN showed additive interactions against *E. faecalis* ATCC 29212 (FICI = 0.787), *E. coli* ATCC 8739 (FICI = 0.577), *E. coli* ATCC 25922 (FICI = 0.940), and *K. pneumoniae* ATCC 13883 (FICI = 0.920).

Overall, the results demonstrate that synergistic and additive interactions are distributed among all the propolis extract combinations. Although the occurrence of these interactions varied according to the microorganism tested, additive interactions were observed across all mixtures, indicating that several extract combinations maintained complementary antimicrobial effects for the predefined concentrations tested in this study.

## 4. Discussion

The present study provides an integrated assessment of the chemical composition, stability, antioxidant and antimicrobial properties of Romanian propolis preparations, with particular emphasis on combinations of extracts from different geographical sources (Supplementary Table S15).

Comparison with the same extracts analysed in 2024 [6] showed that TPC decreased after two years of storage, with reductions ranging from 0.13% in sample A to 29.18% in C2. Similar changes in phenolic content during storage have been reported for plant matrices and bee-product extracts [35,36,37,38]. A multitude of factors may contribute to the decrease in phenolic content observed during storage, including storage time, temperature, and exposure to light, as well as the polarity and specific chemical structure of the individual phenolic compounds present in the extract [37].

Although the TPC of the individual propolis extracts decreased after two years of storage, the mixtures prepared in 2026 showed higher TPC levels when extracts with lower phenolic contents were combined with samples containing higher concentrations of phenolics. For example, sample C2, which exhibited 88.8 mg GAE/g TPC as an individual extract, showed an increase in the phenolic content when mixed with extracts containing higher concentrations of phenolic compounds. Among the C2-based combinations, C2 + S showed the lowest TPC (93.8 mg GAE/g), whereas combinations with C1, H, and A resulted in higher values. Thus, blending C2 with extracts richer in phenolic compounds successfully increased the overall phenolic content of the resulting mixtures (Table 1). This pattern was also observed for mixtures containing extracts with higher TPC values, such as C1 + A and A + H.

Nevertheless, two cases in which the TPC of the mixtures decreased relative to the individual extracts were observed. The combination of C1 (126.5 mg GAE/g) with S (124.8 mg GAE/g) resulted in a TPC of 106.8 mg GAE/g (C1 + S), while the combination of A (149.8 mg GAE/g) with H (147.8 mg GAE/g) resulted in a TPC of 141.9 mg GAE/g. However, these minor reductions do not significantly alter the quality of the resulting extracts. The combination of all five extracts in equal proportions yielded, in most cases, a TPC corresponding to the average of the individual samples.

TFC showed a different temporal pattern. Values increased from 1.5–13.0 mg QE/g in 2024 to 2.4–20.0 mg QE/g in 2026. Changes in the measured TFC may reflect modifications in the chemical composition of the stored extracts and differences in the response of individual flavonoid compounds to the AlCl_3_-based assay. Hydrolysis of flavonoid glycosides during storage may represent one possible chemical process, particularly in the presence of water; however, the present data do not allow the contribution of specific degradation or transformation reactions to be established. The pairwise combinations generally showed TFC values within the range expected from the corresponding individual extracts, whereas PAN showed a lower value than several of the individual preparations. Overall, most of the pairwise mixtures or the five-extract mixture showed a phenolic content similar to the average values of the single PPEs. The observed changes in TPC and TFC during storage, together with the differences among the mixtures, highlight the dynamic and complex nature of the chemical composition of propolis preparations. Therefore, the individual phenolic profiles were further investigated by HPLC to characterise the specific compounds underlying this chemical variability.

Romanian propolis is predominantly of the poplar type, also known as brown propolis. Its composition has been reported to include a diverse range of compounds, mainly phenolic acids and flavonoids [37,38,39,40,41]. Nevertheless, the chemical composition of propolis may differ according to geographical and botanical factors, including the specificity of local flora, harvesting time, and beekeeping practices [39].

In the present study, 19 phenolic acids and flavonoids, together with one phenolic aldehyde and one phenolic acid ester, were identified and quantified in the analysed extracts. All phenolic compounds indicated in the international standard ISO 24381:2023, *Bee propolis–Specifications* [13], for poplar-type propolis were detected in the analysed samples. The individual extracts, regardless of geographical origin, contained apigenin, caffeic acid phenethyl ester (CAPE), caffeic acid, chrysin, ferulic acid, galangin, naringenin, protocatechuic acid, pinocembrin, vanillin, rosmarinic acid, vanillic acid, and p-coumaric acid. However, their concentrations varied considerably, ranging from 0.17 to 391.44 µg/g. Among these compounds, CAPE, chrysin, pinocembrin, ferulic acid, and p-coumaric acid were quantified at the highest levels.

Pinocembrin was present at the highest concentration in samples C1 and H (391.44 and 305.75 µg/g, respectively), whereas chrysin reached the highest concentrations in C1 and C2 (198.14 and 190.71 µg/g, respectively). CAPE was detected at its highest concentrations in C2 and H (157.69 and 238.74 µg/g, respectively). In contrast, catechin, chlorogenic acid, kaempferol, naringin, quercetin, and quercitrin were not detected in any of the five samples originating from different geographical locations. These compounds are not considered reference compounds for poplar-type propolis; therefore, their presence or absence may vary according to the geographical and botanical origin of the resin and is not necessarily expected in every extract.

Analysis of the binary combinations revealed an increase in the concentration of individual phenolic compounds in several cases. The observed changes in the phenolic profile and in the relative contribution of individual compounds may be related to differences in the chemical structures of the phenolic constituents, particularly the position and nature of their functional groups and, consequently, their chemical reactivity. Another factor that may contribute to changes in the phenolic profile over time or following combination is the extraction solvent. In the present study, the extracts were prepared using 80% ethanol, meaning that the remaining 20% water may favour hydrolytic reactions. During prolonged storage, water molecules may contribute to the hydrolysis of ester bonds in complex phenolic structures. For example, caffeic acid phenethyl ester (CAPE) may undergo hydrolysis into simpler compounds, potentially resulting in a decrease in its concentration, while the concentrations of other resulting compounds may increase. Thus, the observed changes in the phenolic profile over time and following the combination of extracts may reflect the interaction between the chemical characteristics of individual compounds and the physicochemical conditions of the extracts.

The HPLC analysis confirmed substantial differences in the phenolic profiles of the individual extracts and their combinations. These differences provide a potential chemical basis for the variation observed in the functional properties of the preparations and prompted further evaluation of their antioxidant activity.

Antioxidant activity differed among preparations and among analytical assays. Extract A showed consistently high DPPH, ABTS, and FRAP values, whereas S showed high FRAP and ORAC values but comparatively lower DPPH activity. Such assay-dependent differences are consistent with previous studies showing that propolis samples can produce different rankings depending on the analytical method used [40,41,42].

The combinations also showed assay-dependent responses. Sample A + S exhibited the highest ORAC value (3213.74 ± 11.47 mg TE/g), significantly exceeding the other preparations, whereas this difference was not observed to the same extent in the DPPH and FRAP assays. Conversely, C1 + C2 and C2 + S showed comparatively low ORAC values. These results indicate that combining extracts did not uniformly increase antioxidant activity and that the response depended on the assay considered. Similar differences among antioxidant assays have been reported for propolis samples with different chemical profiles [40,41,43,44].

Beyond antioxidant activity, the chemical diversity of the propolis preparations may also contribute to differences in their antimicrobial properties. The antimicrobial activity of the individual extracts and their combinations was therefore evaluated against a broad panel of bacterial and yeast strains. The antimicrobial activity of the propolis extracts was strongly dependent on both the extract and the microorganism tested. The inhibition-zone data showed substantial variation among the five individual extracts and their binary combinations. This variability is consistent with the recognised chemical complexity of propolis and the marked influence of botanical and geographical origin, environmental conditions and extraction procedures on its chemical composition and biological properties [16,45,46,47]. More than 800 compounds have been reported in different propolis types, including flavonoids, phenolic acids, terpenes and other phenolic derivatives, and differences in their relative abundance can substantially influence antimicrobial activity [47]. Consequently, the distinct antimicrobial profiles observed among C1, C2, A, S and H are compatible with differences in their underlying chemical composition.

The inhibition-zone assay demonstrated substantial variation among both extracts and microorganisms. Among the Gram-positive bacteria, C1 produced some of the largest inhibition zones, including 22.21 ± 0.50 mm against *S. aureus* ATCC 25923, 19.95 ± 0.64 mm against MRSA ATCC 43300, 20.32 ± 0.44 mm against *S. epidermidis* ATCC 12228, and 21.09 ± 0.01 mm against *L. monocytogenes* ATCC 35152. The enterococci showed a more heterogeneous response, with C1 + S producing 18.76 ± 0.08 mm against *E. faecium*, whereas S + H produced 10.65 ± 0.33 mm against *E. faecalis*. These observations are consistent with previous reports describing substantial differences in propolis susceptibility among Gram-positive species and strains [6,9,45,48,49].

A different pattern was observed among Gram-negative bacteria. S produced relatively large inhibition zones against *P. mirabilis* (19.36 ± 0.35 mm), *A. baumannii* (16.40 ± 0.55 mm), *S. enterica* (21.15 ± 0.08 mm), and *E. coli* ATCC 25922 (15.11 ± 0.38 mm). A + S produced the largest zone against *P. aeruginosa* (19.41 ± 0.24 mm), followed by C2 + S (17.57 ± 0.36 mm). In contrast, *K. pneumoniae* showed comparatively limited inhibition, with C1 producing the largest zone (12.57 ± 0.22 mm). Thus, susceptibility varied substantially among individual Gram-negative species, in agreement with previous studies of Romanian and European propolis [14,45,50].

The two yeast strains also showed different responses. C1 produced the largest inhibition zone observed in the dataset against *C. albicans* (24.73 ± 0.41 mm), while S produced the largest zone against *C. parapsilosis* (20.75 ± 0.43 mm). Several combinations also showed high activity against these organisms. This species-dependent response is consistent with previous evidence that the anticandidal activity of propolis varies among extracts and *Candida* species [51].

The binary combinations did not systematically increase inhibition-zone diameters. For example, A + S produced the largest zone against *P. aeruginosa*, whereas C1 alone produced a larger zone against *S. aureus* ATCC 25923 than C1 + A or C1 + S. Similarly, the inhibition zone produced by S against *P. mirabilis* exceeded those of all S-containing combinations tested against this strain. Thus, the effect of combining extracts varied according to both the combination and the target microorganism.

The absence of a universal enhancement following combination is particularly relevant for chemically complex natural products such as propolis. The biological activity of propolis is associated with a diverse mixture of phenolic acids, flavonoids, terpenes, and other bioactive constituents [16,47]. Consequently, combining chemically distinct extracts may alter the overall composition of the preparation and, in turn, its biological activity. The recent literature has also emphasised that the antimicrobial activity of propolis varies according to both extract characteristics and the target microorganism [46]. Therefore, the inhibition-zone data suggest selective effects of the extract combinations rather than a general potentiation of antimicrobial activity.

Overall, the inhibition-zone assay demonstrated that C1 had the most consistent activity against the tested Gram-positive bacteria and *C. albicans*, whereas S showed comparatively strong activity against several Gram-negative bacteria and *C. parapsilosis*. The distinct response profiles of the individual extracts and their combinations highlight the importance of considering both extract composition and microorganism identity when evaluating propolis antimicrobial activity.

Although the inhibition-zone assay provided an initial assessment of antimicrobial susceptibility, it did not directly quantify the concentration required to inhibit microbial growth. MIC determination therefore provided a complementary concentration-based assessment of the antimicrobial potency of the individual extracts and their combinations. No single preparation displayed the lowest MIC across the entire microbial panel. Instead, C1 was particularly active against several Gram-positive bacteria and *C. albicans*, whereas S showed comparatively low MIC values against selected Gram-negative bacteria and *C. parapsilosis*. Such variability is consistent with the established relationship between propolis composition and antimicrobial activity [16,47].

Among the Gram-positive bacteria, C1 showed particularly strong activity. The lowest MIC values were observed against *S. aureus* ATCC 25923 (0.040 mg/mL) and *S. epidermidis* ATCC 12228 (0.057 mg/mL), while MICs of 0.190 and 0.160 mg/mL were obtained against MRSA ATCC 43300 and *L. monocytogenes* ATCC 35152, respectively. These results are consistent with the inhibition-zone data, where C1 also produced some of the largest zones against the same organisms. The strong susceptibility of *Staphylococcus* spp. to C1 is consistent with previous reports describing *S. aureus* and other *Staphylococcus* species as among the bacterial taxa frequently susceptible to propolis preparations [9,45]. Nevertheless, the higher MIC observed for MRSA relative to *S. aureus* indicates that susceptibility may also vary between strains. The enterococci exhibited a more heterogeneous MIC profile. For *E. faecalis*, MIC values ranged from 0.390 mg/mL for C1 to 6.250 mg/mL for S + H, while those for *E. faecium* ranged from 0.190 mg/mL for C1 + S to 2.080 mg/mL for C1 + C2 and C2 + H. Thus, certain combinations substantially enhanced activity against selected enterococcal strains, most notably C1 + S against *E. faecium*, whereas other combinations resulted in reduced activity. Overall, this pattern further indicates that antimicrobial potency was not consistently related to the number of extracts included in a preparation.

Among the yeasts, *C. albicans* exhibited the greatest susceptibility. C1 produced the lowest MIC observed across the entire dataset (0.027 mg/mL), followed by C2 and S (0.040 mg/mL). Low MIC values were also observed for several combinations, including C1 + S (0.033 mg/mL), C1 + A (0.040 mg/mL), and A + S (0.073 mg/mL). These findings were consistent with the inhibition-zone assay, in which C1 produced the largest inhibition zone against *C. albicans*. In contrast, *C. parapsilosis* generally displayed higher MIC values, although S showed pronounced activity against this strain (0.090 mg/mL), followed by A + S (0.190 mg/mL), C2 + S (0.260 mg/mL), and A + H (0.260 mg/mL).

The pronounced difference in susceptibility between the two *Candida* species is consistent with recent evidence showing substantial variability in the anticandidal activity of ethanolic propolis extracts. Bollin et al. [51], for instance, reported that only 7 of 83 ethanolic propolis extracts (8.4%) exhibited high anticandidal activity and identified several flavonoid constituents, including pinocembrin, pinobanksin-3-acetate, chrysin, and galangin, as potential markers associated with enhanced antifungal activity. Importantly, their study also demonstrated that combinations of selected compounds could exhibit greater antifungal activity than the corresponding individual compounds, highlighting the importance of the overall chemical profile and interactions among constituents in determining the anticandidal activity of propolis [51]. These observations support the hypothesis that differences in the chemical composition of the extracts may contribute to the species-specific responses observed in the present study.

The Gram-negative MIC profiles exhibited a distinct pattern. S showed particularly low MIC values against *S. enterica* ATCC 25928 (0.160 mg/mL), *P. mirabilis* ATCC 29906 (0.190 mg/mL), and *A. baumannii* ATCC 19606 (0.390 mg/mL). A + S displayed the lowest MIC against *P. aeruginosa* ATCC 27853 (0.190 mg/mL), while C2 + S also showed strong activity against this species (0.390 mg/mL). S + H produced low MIC values against *P. mirabilis* (0.260 mg/mL) and *E. coli* ATCC 8739 (0.520 mg/mL). The pronounced activity of S against several Gram-negative organisms was consistent with the inhibition-zone assay and with previous studies showing that propolis extracts can display substantial activity against Gram-negative pathogens. However, the magnitude of this activity varies considerably according to both the extract and the microorganism. In a recent study specifically examining Romanian propolis, ethanolic extracts exhibited measurable activity against *P. aeruginosa*, *E. coli*, *S. enterica*, and *A. baumannii*, with MIC values differing among extracts and bacterial species [50]. Similar variability among Gram-negative species has also been reported for European propolis extracts, including differences in susceptibility among *E. coli*, *P. aeruginosa*, *K. pneumoniae*, *P. mirabilis*, *Salmonella*, and *A. baumannii* [14,45].

*K. pneumoniae* exhibited comparatively high MIC values across the treatment panel, ranging from 1.560 for C1 to 8.330 mg/mL for C1 + C2. The latter represented the highest MIC recorded among all propolis preparations tested. This finding indicates that combining two extracts with measurable antimicrobial activity does not necessarily result in enhanced inhibitory potency. Similar treatment-dependent differences were observed between the two *E. coli* strains. S produced MIC values of 4.160 and 0.780 mg/mL against *E. coli* ATCC 8739 and ATCC 25922, respectively, whereas C1 + S produced MIC values of 0.780 and 5.210 mg/mL against the same strains. Thus, even within a single bacterial species, the activity of a given extract or combination was strongly dependent on the strain.

Comparison of the individual extracts with their corresponding combinations further highlighted the selective nature of the combination effects. A + S produced an MIC of 0.190 against *P. aeruginosa*, compared with 1.300 mg/mL for A and 0.780 mg/mL for S, indicating an apparent enhancement of activity against this specific target. C1 + H similarly produced an MIC of 0.073 mg/mL against *S. epidermidis*, compared with 0.057 mg/mL for C1 and 0.390 mg/mL for H. In contrast, C1 + C2 resulted in an MIC of 8.330 mg/mL against *K. pneumoniae*, compared with 1.560 mg/mL for C1 and 4.160 mg/mL for C2. C1 + S also resulted in a marked increase in MIC against *E. coli* ATCC 25922 (5.210 mg/mL) relative to C1 (2.600 mg/mL) and S (0.780 mg/mL). Taken together, these contrasting responses indicate that the effect of a combination depends on both the specific extract pair and the target microorganism, rather than simply reflecting an additive contribution of the individual extracts. Such variability is consistent with previous investigations showing that the antimicrobial activity of propolis can differ substantially according to the extract, microorganism, and combination under evaluation [14,52].

This behaviour can be considered in the context of the complex chemistry of propolis. Propolis contains numerous classes of bioactive constituents, and its chemical composition can vary substantially according to geographical and botanical origin, season, and extraction conditions, potentially affecting its biological properties [47,53,54]. The presence of several active extracts within a mixture therefore does not necessarily guarantee greater antimicrobial potency, as differences in chemical composition may alter the overall biological response. Experimental studies have demonstrated that interactions involving propolis can vary according to the specific compounds or antimicrobial agents combined, with both enhanced and antagonistic effects reported under different experimental conditions [52,55]. These observations support the view that the antimicrobial activity of propolis results from the collective contribution of multiple constituents rather than from a single universally dominant compound.

Importantly, the MIC results obtained in the present study should not, by themselves, be interpreted as evidence of synergy. Rather, they identify specific extract–microorganism combinations in which enhanced or reduced activity was observed and for which a formal interaction analysis is warranted. Synergistic, additive, indifferent, or antagonistic interactions require an appropriate combination assay and quantitative criterion, such as the fractional inhibitory concentration index (FICI). This distinction is important because apparent enhancement or reduction in MIC values does not necessarily correspond to a formally defined synergistic or antagonistic interaction [14,55].

The control treatments further support the interpretation of the observed antimicrobial activity. The 80% ethanol control showed MIC values >50 for all microorganisms, whereas the propolis preparations exhibited substantially lower MIC values. Under the conditions of the present assay, this indicates that the observed antimicrobial activity cannot be attributed simply to the ethanol vehicle. The reference antibiotics likewise showed markedly lower MIC values against their respective bacterial targets, providing an appropriate benchmark against which the activity of the propolis preparations could be evaluated.

Taken together, the MIC results confirmed that antimicrobial activity was strongly dependent on both the propolis preparation and the target microorganism, with selected combinations showing enhanced activity but no universal improvement across the microbial panel. Given the multidimensional nature of the chemical and biological datasets, multivariate analyses were subsequently performed to explore whether variation in chemical composition was associated with the observed antimicrobial profiles.

The multivariate analyses indicate that differences in the chemical composition of the propolis extracts were associated with distinct biological-response profiles. The chemical PCA demonstrated differences among individual extracts and combinations, reflecting variation in phenolic compounds and antioxidant-related variables. The biological PCA similarly indicated that antimicrobial responses varied across microorganisms and response measures. A significant inverse correlation was observed between chemical PC1 and biological PC2 (ρ = −0.809, adjusted *p* = 0.0013), indicating an association between a major axis of chemical variation and a major axis of antimicrobial variation. Because both variables represent composite principal components, this association cannot be attributed to any individual compound.

While PCA provided an overview of the major patterns of variation within the chemical and biological datasets, PLS analysis was subsequently used to identify the variables that contributed most strongly to these multivariate patterns. The exploratory PLS analysis provided further insight into the chemical variables associated with biological variation. Because the PLS analysis was exploratory and no formal inferential significance testing was applied to individual predictors, VIP values were used to characterise variable importance rather than statistical significance [56]. Rosmarinic acid, ABTS, catechin, pinocembrin, chlorogenic acid, naringenin, DPPH, and quercetin showed VIP values >1, identifying them as relatively influential predictors within the model. The recurrence of phenolic compounds among the highest-ranking variables suggests that variation in specific components of the phenolic profile, rather than total phenolic abundance alone, may contribute to differences in biological responses. This interpretation is consistent with the recognised chemical complexity of propolis and with previous evidence linking differences in phenolic composition to variation in its biological properties [8,45,46,57]. Nevertheless, VIP values represent variable importance within a multivariate model and should not be interpreted as measures of statistical significance or causality. Although VIP > 1 is widely used as a practical criterion for identifying influential predictors, formal assessment of VIP significance requires dedicated inferential procedures [56]. In a complex matrix such as propolis, chemically correlated constituents may contribute jointly to the observed response [57]. Thus, the present findings identify candidate chemical features associated with antimicrobial variability rather than definitive antimicrobial markers.

Overall, the PCA and exploratory PLS analyses indicate that the chemical composition of propolis is associated with its antimicrobial profile, while emphasising that this relationship reflects the combined effects of multiple constituents. These findings complement the direct antimicrobial assays and identify chemical variables for further targeted investigation.

The multivariate analyses therefore provided evidence of an association between chemical composition and antimicrobial response, but did not directly determine whether the observed effects of combining extracts resulted from synergistic, additive, indifferent, or antagonistic interactions. To address this question, the binary combinations were further evaluated using the fractional inhibitory concentration index (FICI). Although the ability of propolis to participate in synergistic interactions with other substances, such as antibiotics and antimycotics, has been established in the literature [14,46], the present study specifically examined interactions among different PPE combinations. The results are consistent with previous studies while providing information on inter-extract compatibility and combinatorial activity between extracts [14,46], while also supporting recent literature concerning propolis activity and its potential contribution to standardisation [17,18]. Among the 176 determinations, only nine resulted in antagonistic interactions, which may be associated with differences in chemical composition, concentration-dependent effects, or interactions among compounds.

The combinations of PEEs can produce different interaction classes depending on the biological context. Overall, the extracts appear broadly compatible, whereas synergistic interactions were selective rather than universal. The occurrence of synergistic and additive interactions is consistent with the complex phytochemical composition of propolis and the proposed contribution of multiple compounds acting through complementary antimicrobial mechanisms. Previous studies have demonstrated synergistic interactions between PEEs and conventional antibacterial or antifungal agents, although the magnitude and direction of these interactions depend strongly on propolis origin, chemical composition, the antimicrobial agent, and the target microorganism [8,14,58]. The high proportion of indifferent interactions observed in the present study may therefore reflect overlapping antimicrobial effects among the different propolis extracts rather than a loss of activity. Importantly, antagonistic interactions represented only 5.11% of the observations, indicating that detrimental interactions were relatively uncommon against the studied organisms. Overall, these results support the potential for selected propolis extract combinations to produce additive or synergistic antimicrobial effects, while indicating that such interactions are selective rather than universal.

From the perspective of synergistic interactions, the A + S combination presented the broadest synergistic profile, particularly against Gram-negative bacteria, while A + H showed synergistic interactions against *E. faecalis* ATCC 29212, *A. baumannii* ATCC 19606, *S. enterica* ATCC 25928, and *S. typhimurium* ATCC 14028 (Supplementary Table S13). The C2 + A, C2 + H, and S + H combinations also showed synergistic activity against *S. aureus* MRSA ATCC 43300, whereas C1 + S, C2 + S, A + S, and S + H showed synergistic interactions against *E. coli* ATCC 8739.

The C1 + C2 combination was the most notable from the perspective of antagonistic interactions, accounting for four of the nine antagonistic interactions observed against *E. faecalis* ATCC 29212, *E. faecium* ATCC 19434, *A. baumannii* ATCC 19606, and *S. typhimurium* ATCC 14028. Furthermore, two antagonistic interactions were observed for C1 + S against *C. parapsilosis* ATCC 22019 and *E. coli* ATCC 25922, while two were observed for PAN against *C. albicans* ATCC 10231 and *A. baumannii* ATCC 19606. The C2 + A combination showed one antagonistic interaction against *P. mirabilis* ATCC 29906. Thus, antagonistic interactions among the analysed mixtures were associated with specific microorganisms rather than representing a general characteristic of a particular propolis combination. Overall, combining propolis extracts did not generally result in mutual inhibition of their antimicrobial effects, which is consistent with previous reports concerning propolis biocompatibility [46,59]. This variability is also consistent with studies examining propolis in combination with antimicrobial agents, in which favourable interactions, including additive and synergistic effects, as well as antagonistic interactions, have been reported [17,18,55,59].

Regarding the predominance of indifferent interactions across the extract combinations, the results indicate that the resulting combinations generally retained their antimicrobial activity, with relatively few interactions associated with a measurable reduction in antimicrobial performance. This observation is also consistent with the complex chemical composition of propolis extracts, which contain multiple compounds that may contribute to their biological activity [9,60]. The PAN mixture, which contained all five extracts in equal proportions, did not result in stronger interactions compared with the binary mixtures. Thus, increasing the number of extracts in a mixture did not necessarily increase the occurrence of synergistic or additive interactions. Combining a larger number of extracts may increase chemical diversity without necessarily increasing antimicrobial activity, as also suggested by previous studies [57,61].

From the perspective of the microorganism response, a marked dependence of the interaction outcome on the target microorganisms was observed. The same propolis combination could show a synergistic interaction against one microorganism, while exhibiting an additive, indifferent, or, in some cases, antagonistic interaction against another. This response may depend not only on the chemical composition of the mixture but also on the physiological characteristics of the microorganism. For example, the sensitivity of *S. aureus* to propolis is well documented [9,52]. In the present study, however, the mixtures produced three additive and three synergistic interactions among the 11 combinations tested against *S. aureus* MRSA ATCC 43300, whereas only three additive interactions were observed for *S. aureus* ATCC 25923. Differences in the adaptation or physiological characteristics of individual strains may contribute to differences in their sensitivity to propolis [6,62], which may help explain the different interaction profiles observed.

It was also observed that favourable interactions were less frequent among tested yeasts, with most favourable interactions occurring against Gram-positive and Gram-negative bacteria. The higher number of synergistic and additive responses observed for Gram-negative bacteria suggests a potentially favourable interaction profile for some binary mixtures. One of the clearest examples was *E. coli* ATCC 8739, for which four synergistic and five additive interactions were observed, together with three indifferent interactions. Similarly, *K. pneumoniae* ATCC 13883 showed six additive and one synergistic interaction. In contrast, *P. mirabilis* ATCC 29906 showed one antagonistic and ten indifferent interactions, indicating a lower responsiveness to the combinations tested. These findings indicate that some propolis combinations may have potential for further investigation in the context of antimicrobial applications, including against antibiotic-resistant strains.

Despite the inclusion of only two yeast strains, one synergistic and two antagonistic interactions were observed, with the remaining interactions classified as indifferent. This indicates that the combinations tested in the present study had limited effects on the antifungal activity against the tested strains. The limited number of fungal strains represents a limitation of the study; therefore, further research including a broader range of fungal strains would be useful to better characterise the potential of propolis extract combinations against fungal targets.

Taken together, the FICI results further demonstrate that the effects of combining propolis extracts for the predefined ratios are highly dependent on both the composition of the combination and the target microorganism. When considered alongside the chemical, antioxidant, antimicrobial and multivariate analyses, these findings indicate that combining propolis extracts modifies their functional properties in a selective rather than uniformly enhancing manner.

## 5. Conclusions

The present study demonstrates substantial variability in the chemical composition and biological activity of Romanian propolis single extract and mixtures. TPC and TFC analyses revealed storage-related changes over two years, while HPLC confirmed distinct phenolic profiles among individual extracts and their combinations, highlighting the chemical heterogeneity of propolis. Antioxidant activity was strongly assay-dependent, with no preparation consistently showing the highest activity across all assays. Notably, A + S exhibited the highest ORAC activity. Antimicrobial activity and MIC values also varied according to both propolis extracts and the target microorganism, and combining extracts did not systematically enhance antimicrobial potency.

PCA further indicated an association between chemical composition and biological responses, while FICI analysis showed that interactions between extracts were predominantly indifferent, with selected synergistic, additive and a total of nine antagonistic effects. Importantly, interaction outcomes depended on both the extract combination and the target microorganism.

Overall, these findings indicate that combining propolis extracts does not simply increase their chemical or biological activity, but can modify their functional properties in a target-dependent manner. The identification of combinations with enhanced antioxidant or antimicrobial activity highlights their potential for further investigation, while the occurrence of indifferent and antagonistic interactions emphasises the need for systematic chemical characterisation and interaction testing. Rather than supporting the assumption that combining a greater number of propolis sources will necessarily produce a more active preparation, the present results suggest that the selection of specific extracts and their chemical compatibility should be considered when developing standardised propolis-based preparations for functional or antimicrobial applications.

## Figures and Tables

**Table 1:** Total phenolic content and total flavonols of the 1% PEEs. The data were expressed as the mean ± SD (*n* = 3). Different lowercase letters (a–m) in each column indicate significant differences, *p* < 0.05.

Sample ID	TPC	TFC
C1	126.5 ± 0.3 ^f^	20 ± 0.4 ^a^
C2	88.8 ± 0.3 ^m^	19.9 ± 0.1 ^a^
A	149.8 ± 0.5 ^a^	16.5 ± 0.5 ^b^
S	124.8 ± 0.5 ^g^	5.4 ± 0.2 ^g^
H	147.8 ± 0.8 ^b^	2.4 ± 0.4 ^i^
PAN	123.3 ± 0.4 ^h^	9 ± 0.7 ^de^
C1 + C2	113.0 ± 0.4 ^j^	13.5 ± 0.4 ^c^
C1 + A	136.0 ± 0.8 ^d^	15.6 ± 0.8 ^b^
C1 + S	106.8 ± 0.5 ^k^	9.1 ± 0.4 ^de^
C1 + H	128.7 ± 0.2 ^e^	8.4 ± 0.7 ^ef^
C2 + A	114.8 ± 0.3 ^i^	12.9 ± 0.8 ^c^
C2 + S	93.8 ± 0.7 ^l^	8.8 ± 0.1 ^e^
C2 + H	114.2 ± 0.5 ^ij^	7.1 ± 0.4 ^f^
A + S	128.1 ± 0.5 ^e^	12.3 ± 0.6 ^c^
A + H	141.9 ± 0.5 ^c^	10.1 ± 0.1 ^d^
S + H	143.2 ± 0.5 ^c^	4 ± 0.2 ^h^

Total Phenolic Compound (TPC): mg Gallic Acid Equivalent (GAE)/g sample; Total Flavonoid Content (TFC): mg quercetin equivalents (QE)/g sample.

**Table 2:** Antioxidant activity of the propolis ethanolic extracts. The data are expressed as mean ± SD (*n* = 4). Different lowercase letters (a–k) in each column indicate significant differences (*p* < 0.05).

	DPPH	ABTS	FRAP	ORAC
C1	28.5 ± 0.4 ^bc^	35.9 ± 0.3 ^ab^	6.4 ± 0.6 ^bcde^	2906.64 ± 12.76 ^c^
C2	19.0 ± 0.6 ^g^	28.4 ± 0.6 ^g^	5.6 ± 0.3 ^defg^	2194.51 ± 16.70 ^k^
A	29.7 ± 0.1 ^ab^	36.5 ± 0.3 ^a^	7.6 ± 0.3 ^a^	2755.21 ± 13.63 ^f^
S	7.9 ± 0.5 ^g^	31.1 ± 0.7 ^de^	7.2 ± 0.5 ^ab^	2881.06 ± 19.04 ^cd^
H	22.9 ± 0.5 ^e^	32.1 ± 0.7 ^d^	6.5 ± 0.4 ^bcd^	2970.77 ± 21.61 ^b^
PAN	26.0 ± 0.6 ^d^	34.9 ± 0.4 ^bc^	5.6 ± 0.3 ^efg^	2736.07 ± 10.85 ^f^
C1 + C2	20.7 ± 0.9 ^f^	35.6 ± 0.6 ^abc^	5.4 ± 0.1 ^fgh^	2206.01 ± 16.71 ^k^
C1 + A	28.6 ± 0.5 ^bc^	30.5 ±0.5 ^e^	6.3 ± 0.4 ^cde^	2625.67 ± 12.30 ^g^
C1 + S	28.3 ± 0.4 ^c^	28.8 ± 0.5 ^fg^	5.2 ± 0.2 ^gh^	2737.23 ± 12.36 ^f^
C1 + H	30.3 ± 0.5 ^a^	32.3 ± 0.3 ^d^	6.2 ± 0.3 ^cdef^	2446.49 ± 16.51 ^i^
C2 + A	30.0 ± 0.4 ^a^	34.3 ± 0.7 ^c^	5.6 ± 0.3 ^defg^	2503.86 ± 14.18 ^h^
C2 + S	27.4 ± 0.7 ^c^	28.5 ± 0.8 ^fg^	4.6 ± 0.3 ^h^	2288.51 ± 15.48 ^j^
C2 + H	27.9 ± 0.3 ^c^	29.9 ± 0.5 ^ef^	5.2 ± 0.3 ^gh^	2860.91 ± 10.77 ^d^
A + S	29.6 ± 0.4 ^ab^	35.9 ± 0.7 ^ab^	6.2 ± 0.3 ^cde^	3213.74 ± 11.47 ^a^
A + H	30.5 ± 0.2 ^a^	35.4 ± 0.3 ^abc^	6.6 ± 0.3 ^bc^	2615.99 ± 17.81 ^g^
S + H	25.0 ± 0.7 ^d^	30.5 ± 0.7 ^e^	5.9 ± 0.3 ^cdefg^	2880.08 ± 16.25 ^cd^
PC	78.9 ± 0.7	50.5 ± 0.8	59.3 ± 0.5	32.60 ± 0.35

2,2-diphenyl-1-picrylhydrazyl (DPPH), 2,2′-azino-bis(3-ethylbenzothiazoline-6-sulfonic acid (ABTS), Ferric Reducing Antioxidant Power (FRAP), and Oxygen Radical Absorbance Capacity (ORAC) are expressed as mg Trolox equivalents (TE)/g.

## Data Availability

All data supporting the results of this study are provided in the current paper and in the Supplementary Materials. Raw data and additional information are available from the corresponding authors upon reasonable request.

## Supplementary Materials

The following supporting information can be downloaded at https://www.mdpi.com/article/10.3390/antibiotics15100972/s1: Table S1. Phenolic profiles of the propolis ethanolic extracts; Table S2. Antimicrobial activity of individual propolis ethanolic extracts (PEEs) and their binary combinations against Gram-positive bacterial strains and yeasts, determined by the agar well diffusion assay; Table S3. Antimicrobial activity of individual propolis ethanolic extracts (PEEs) and their binary combinations against Gram-negative bacterial strains, determined by the agar well diffusion assay; Table S4. Minimum inhibitory concentration (MIC) values of individual propolis ethanolic extracts (PEEs) and their binary combinations against Gram-positive bacterial strains and yeasts; Table S5. Minimum inhibitory concentration (MIC) values of individual propolis ethanolic extracts (PEEs) and their binary combinations against Gram-negative bacterial strains and yeasts; Table S6. Eigenvalues and percentage of variance explained by the first principal components obtained from the biological principal component analyses (PCA); Table S7. Loadings, contributions, and squared cosine values (cos^2^) of the antimicrobial activity and MIC variables for the first three principal components (PC1–PC3) obtained from the biological dataset; Table S8. Loadings, contributions, and squared cosine values (cos^2^) of the biological variables on the first three principal components (PC1–PC3); Table S9. Eigenvalues and percentage of variance explained by the first principal components obtained from the chemical principal component analyses (PCA); Table S10. Loadings, contributions, and squared cosine values (cos^2^) of the antimicrobial activity and MIC variables for the first three principal components (PC1–PC3) obtained from the chemical dataset; Table S11. Loadings, contributions, and squared cosine values (cos^2^) of the chemical variables on the first three principal components (PC1–PC3); Table S12. Spearman correlation coefficients between the first three principal components (PC1–PC3) derived from the chemical and biological principal component analyses (PCAs); Table S13. FICI values and interaction profiles of binary propolis ethanolic extract combinations against Gram-positive bacterial and yeast strains; Table S14. FICI values and interaction types of binary propolis ethanolic extract combinations against Gram-negative bacterial strains; Table S15. Geographic location of the apiaries from which propolis samples were collected. Reference [6] is cited in the Supplementary Materials.
