# Supplementary material for: Antioxidant and Antimicrobial Potential of Romanian Propolis Extract Combinations: Linking Chemical Composition with Biological Activity
Source: Antibiotics (Basel). 2026 Oct 1;15(10):972. doi: 10.3390/antibiotics15100972 (PMC13700676; doi:10.3390/antibiotics15100972)
Supplement: Supplementary file 1 [file antibiotics-15-00972-s001.zip › Supplementary_table15.pdf]

Table S15. Geographic location of the apiaries from which propolis samples were collected.

	County	Apiary
C1	Cluj	USAMV apiary
C2	Cluj	Palocsay apiary
A	Alba	Alba apiary
S	Sălaj	Sălaj apiary
H	Harghita	Corund apiary

Adapted from Albanese et al. (2025) [1].

References

1. Albanese, G.; Giurgiu, A.I.; Bobiș, O.; Urcan, A.C.; Botezan, S.; Bonta, V.; Ternar, T.N.; Pașca, C.; Iorizzo, M.; De Cristofaro, A.; et al. Functional and Antimicrobial Properties of Propolis from Different Areas of Romania. *Applied Sciences* **2025**, *15*, 898, doi:10.3390/app15020898.
