# Supplementary material for: Antioxidant and Antimicrobial Potential of Romanian Propolis Extract Combinations: Linking Chemical Composition with Biological Activity
Source: Antibiotics (Basel). 2026 Oct 1;15(10):972. doi: 10.3390/antibiotics15100972 (PMC13700676; doi:10.3390/antibiotics15100972)
Supplement: Supplementary file 1 [file antibiotics-15-00972-s001.zip › Supplementary materials complete 6-12.pdf]

1. Biological Dataset

Table S6. Eigenvalues and percentage of variance explained by the first principal components obtained from the biological principal component analyses (PCA).

	Eigenvalue	Variance (%)	Cumulative Variance (%)
PC1	8,938	29,795	29,795
PC2	7,305	24,353	54,148
PC3	3,959	13,199	67,348
PC4	2,331	7,772	75,120
PC5	1,730	5,766	80,887

Table S7. Loadings, contributions, and squared cosine values (cos^2) of the antimicrobial activity and MIC variables for the first three principal components (PC1–PC3) obtained from the biological dataset.

Variable	PC1	PC2	PC3	Contribution PC1	Contribution PC2	Contribution PC3	cos^2 PC1	cos^2 PC2	cos^2 PC3	Variable type
S. aureus MRSA ATCC 43300	-0,545	-0,637	-0,255	3,325	5,549	1,648	0,317	0,432	0,070	AA
S. aureus ATCC 25923	-0,409	-0,718	-0,340	1,871	7,056	2,918	0,178	0,550	0,123	AA
S. epidermidis ATCC 12228	-0,432	-0,624	0,413	2,090	5,327	4,311	0,199	0,415	0,182	AA
E. faecalis ATCC 29212	-0,126	-0,559	-0,592	0,177	4,277	8,855	0,017	0,333	0,374	AA
E. faecium ATCC 19434	0,433	-0,571	0,130	2,101	4,467	0,424	0,200	0,348	0,018	AA
L. monocytogenes ATCC 35152	0,398	-0,761	0,127	1,768	7,924	0,405	0,169	0,617	0,017	AA
E. coli ATCC 8739	0,210	-0,075	-0,307	0,494	0,077	2,376	0,047	0,006	0,100	AA
E. coli ATCC 25922	0,331	-0,057	0,672	1,229	0,045	11,395	0,117	0,003	0,481	AA
P. aeruginosa ATCC 27853	0,638	-0,195	0,122	4,551	0,519	0,378	0,434	0,040	0,016	AA
K. pneumoniae ATCC 13883	-0,127	-0,197	-0,613	0,181	0,530	9,497	0,017	0,041	0,401	AA
P. mirabilis ATCC 29906	0,805	0,318	0,065	7,255	1,383	0,107	0,692	0,108	0,005	AA
A. baumanii ATCC 19606	0,807	-0,275	-0,219	7,277	1,036	1,212	0,694	0,081	0,051	AA
S. enterica ATCC 25928	0,851	-0,029	0,316	8,107	0,011	2,527	0,773	0,001	0,107	AA
S. thiphymurium ATCC 14028	0,828	-0,013	-0,224	7,677	0,002	1,271	0,732	0,000	0,054	AA
C. albicans ATCC 10231	-0,002	-0,800	0,218	0,000	8,761	1,204	0,000	0,683	0,051	AA
C. parapsilopsis ATCC 22019	0,896	-0,222	-0,006	8,987	0,672	0,001	0,857	0,052	0,000	AA
S. aureus MRSA ATCC 43300 MIC	0,488	0,649	0,060	2,665	5,763	0,091	0,254	0,449	0,004	MIC

S. aureus ATCC 25923	0,415	0,776	0,161	1,927	8,236	0,653	0,184	0,642	0,028	MIC
S. epidermidis ATCC 12228	0,340	0,640	-0,414	1,291	5,610	4,333	0,123	0,437	0,183	MIC
E. faecalis ATCC 29212	0,128	0,510	0,572	0,185	3,566	8,250	0,018	0,278	0,348	MIC
E. faecium ATCC 19434	-0,561	0,551	0,114	3,524	4,149	0,329	0,336	0,323	0,014	MIC
L. monocytogenes ATCC 35152	-0,119	0,859	-0,274	0,159	10,092	1,893	0,015	0,786	0,080	MIC
E. coli ATCC 8739	0,230	-0,131	0,334	0,591	0,234	2,815	0,056	0,018	0,119	MIC
E. coli ATCC 25922	0,079	0,057	-0,659	0,070	0,045	10,969	0,007	0,003	0,463	MIC
P. aeruginosa ATCC 27853	-0,606	0,324	-0,113	4,107	1,439	0,322	0,392	0,112	0,014	MIC
K. pneumoniae ATCC 13883	-0,129	0,167	0,542	0,185	0,380	7,405	0,018	0,030	0,313	MIC
P. mirabilis ATCC 29906	-0,693	-0,241	0,247	5,377	0,795	1,542	0,513	0,062	0,065	MIC
A. baumanii ATCC 19606	-0,754	0,225	0,287	6,357	0,691	2,073	0,606	0,054	0,088	MIC
S. enterica ATCC 25928	-0,719	0,169	-0,262	5,789	0,389	1,731	0,552	0,030	0,073	MIC
S. thiphymurium ATCC 14028	-0,639	-0,084	0,534	4,575	0,096	7,210	0,436	0,007	0,305	MIC
C. albicans ATCC 10231	0,010	0,791	-0,269	0,001	8,569	1,826	0,000	0,668	0,077	MIC
C. parapsilopsis ATCC 22019	-0,739	0,411	0,034	6,106	2,312	0,029	0,582	0,180	0,001	MIC

AA: Antimicrobial activity.

Table S8. Loadings, contributions, and squared cosine values (cos²) of the biological variables on the first three principal components (PC1–PC3).

**Sample**	**PC1**	**PC2**	**PC3**	**cos^2 PC1**	**cos^2 PC2**	**cos^2 PC3**	**Group**
C1	-0,528	-4,772	-3,152	0,007	0,603	0,263	Individual
C2	-1,062	-0,598	-2,413	0,059	0,019	0,305	Individual
A	-1,022	-0,392	0,936	0,093	0,014	0,078	Individual
S	6,410	0,073	4,026	0,605	0,000	0,238	Individual
H	0,538	7,189	-1,499	0,005	0,848	0,037	Individual
PAN	-1,755	1,633	-1,411	0,146	0,126	0,094	Mix
C1+C2	-5,098	0,029	3,943	0,521	0,000	0,312	Mix
C1+A	-2,653	-1,908	0,363	0,374	0,193	0,007	Mix
C1+S	2,176	-2,257	-1,570	0,214	0,230	0,111	Mix
C1+H	-1,894	-1,707	1,046	0,199	0,161	0,061	Mix
C2+A	-3,020	-1,774	1,669	0,419	0,145	0,128	Mix
C2+S	2,817	-1,079	-0,142	0,519	0,076	0,001	Mix
C2+H	-3,314	3,910	-0,632	0,260	0,363	0,009	Mix
A+S	4,077	-1,275	0,031	0,579	0,057	0,000	Mix
A+H	1,552	0,437	-1,845	0,152	0,012	0,215	Mix
S+H	2,773	2,490	0,649	0,263	0,212	0,014	Mix

2. Chemical Dataset

Table S9. Eigenvalues and percentage of variance explained by the first principal components obtained from the chemical principal component analyses (PCA).

	**Eigenvalue**	**Variance (%)**	**Cumulative Variance (%)**
PC1	9,838	40,362	40,362
PC2	6,538	26,821	67,183
PC3	2,550	10,460	77,644
PC4	1,724	7,071	84,715
PC5	1,398	5,735	90,450

Table S10. Loadings, contributions, and squared cosine values (cos²) of the antimicrobial activity and MIC variables for the first three principal components (PC1–PC3) obtained from the chemical dataset.

Variable	**PC1**	**PC2**	**PC3**	**Contribution PC1**	**Contribution PC2**	**Contribution PC3**	**Cos² PC1**	**Cos² PC2**	**Cos² PC3**
TPC (mg GAE/g)	0,364	0,749	0,432	1,346	8,587	7,333	0,141	0,599	0,199
TFC (mg QE/g)	0,407	-0,710	-0,355	1,683	7,717	4,946	0,177	0,538	0,135
FRAP (Trolox Eq./g)	0,404	0,732	0,176	1,661	8,205	1,212	0,174	0,572	0,033
DPPH (Trolox Eq./g)	0,804	-0,052	0,168	6,567	0,042	1,112	0,689	0,003	0,030
ABTS (Trolox Eq./g)	0,779	0,203	0,402	6,165	0,630	6,347	0,647	0,044	0,173
ORAC (μmol TE/g)	0,119	0,546	0,737	0,145	4,565	21,275	0,015	0,318	0,579
Protocatechuic acid	0,904	-0,145	0,100	8,304	0,322	0,392	0,871	0,022	0,011
p-Hydroxybenzoic acid	-0,541	0,726	-0,060	2,977	8,065	0,143	0,312	0,562	0,004
Catechin	-0,474	-0,379	-0,213	2,285	2,194	1,774	0,240	0,153	0,048
Vanillic acid	-0,081	0,875	0,203	0,067	11,721	1,618	0,007	0,817	0,044
Chlorogenic acid	-0,415	0,390	0,118	1,747	2,329	0,544	0,183	0,162	0,015
Caffeic acid	0,347	0,713	-0,300	1,223	7,770	3,528	0,128	0,542	0,096
Vanillin	-0,580	0,565	-0,163	3,421	4,888	1,038	0,359	0,341	0,028
p-Coumaric acid	-0,532	0,665	0,090	2,873	6,757	0,318	0,301	0,471	0,009
Ferulic acid	-0,769	0,352	0,069	6,011	1,895	0,187	0,631	0,132	0,005
Naringin	0,705	-0,103	0,306	5,059	0,162	3,670	0,531	0,011	0,100
Rosmarinic acid	-0,578	0,302	0,134	3,402	1,393	0,706	0,357	0,097	0,019
Quercitrin	0,421	-0,499	0,613	1,802	3,804	14,733	0,189	0,265	0,401
Quercetin	0,595	0,392	-0,453	3,598	2,346	8,043	0,378	0,164	0,219
Naringenin	0,860	-0,176	0,187	7,510	0,476	1,375	0,788	0,033	0,037
Apigenin	0,442	0,631	-0,469	1,988	6,085	8,613	0,209	0,424	0,234
Kaempferol	0,757	0,094	-0,207	5,830	0,136	1,682	0,612	0,009	0,046
CAPE	0,638	0,564	-0,265	4,138	4,871	2,757	0,434	0,340	0,075
Chrysin	0,844	0,338	-0,270	7,249	1,746	2,865	0,761	0,122	0,078
Pinocembrin	0,820	-0,165	0,052	6,840	0,416	0,107	0,718	0,029	0,003
Galangin	0,775	0,434	-0,306	6,113	2,878	3,683	0,641	0,201	0,100

Table S11. Loadings, contributions, and squared cosine values (cos^2) of the chemical variables on the first three principal components (PC1–PC3).

Sample	PC1	PC2	PC3	Group
C1	4,94468979	-4,47187233	3,8812975	Individual
C2	1,55274014	-2,47689254	0,44751926	Individual
A	-0,41934616	-2,47279499	-0,50019172	Individual
S	-3,454856	-3,0518015	-0,49015636	Individual
H	-4,88005884	-4,77735529	-1,81427604	Individual
PAN	-0,77929154	0,38418512	-2,39418823	Mix
C1+C2	4,13929815	-0,9228179	-2,01190045	Mix
C1+A	4,67700547	1,92157189	-2,19435639	Mix
C1+S	2,39709546	2,336249	0,30907214	Mix
C1+H	2,67935015	1,89865662	0,36348522	Mix
C2+A	1,9235037	1,50446838	-0,25278233	Mix
C2+S	-1,59166041	1,18859915	1,01441958	Mix
C2+H	-2,33911608	0,81809502	0,7578473	Mix
A+S	-1,91454333	3,00155381	0,10392735	Mix
A+H	-2,31267019	2,5255373	0,55858986	Mix
S+H	-4,62214029	2,59461825	2,22169332	Mix

3. Chemical Biological correlation

Table S12. Spearman correlation coefficients between the first three principal components (PC1–PC3) derived from the chemical and biological principal component analyses (PCAs). Raw *p*-values and Benjamini–Hochberg-adjusted *p*-values are reported.

Chemical PC	Biological PC	Spearman's ϱ	*p*-value	Adjusted *p*-value
PC1	PC1	-0,50294118	0,04706314	0,21178413
PC1	PC2	-0,80882353	0,00014848	0,00133628
PC1	PC3	-0,01470588	0,95689192	0,95689192
PC2	PC1	0,16470588	0,54214814	0,81073272
PC2	PC2	-0,18529412	0,49205705	0,81073272
PC2	PC3	0,09705882	0,72065131	0,81073272
PC3	PC1	0,31176471	0,23980005	0,53955011
PC3	PC2	-0,10882353	0,68828829	0,81073272
PC3	PC3	-0,38529412	0,14054418	0,42163253
