# Supplementary material for: Antioxidant and Antimicrobial Potential of Romanian Propolis Extract Combinations: Linking Chemical Composition with Biological Activity
Source: Antibiotics (Basel). 2026 Oct 1;15(10):972. doi: 10.3390/antibiotics15100972 (PMC13700676; doi:10.3390/antibiotics15100972)
Supplement: Supplementary file 1 [file antibiotics-15-00972-s001.zip › Supplementary_table 2_and 3Antimicrobial_FINAL.pdf]

Table S2. Antimicrobial activity of individual propolis ethanolic extracts (PEEs) and their binary combinations against Gram-positive bacterial strains and yeasts, determined by the agar well diffusion assay, expressed in millimeters (mm). Data are expressed as the mean ± SD (n = 3). Different lowercase letters (a–i) in each column indicate significant differences, $p < 0.05$.

Treatment group	Gram-positive Strains						Yeasts	
	S. aureus MRSA ATCC 43300	*S. aureus* ATCC 25923	*S. epidermidis* ATCC 12228	*E. faecalis* ATCC 29212	*E. faecium* ATCC 19434	*L. monocytogenes* ATCC 35152	*C. albicans* ATCC 10231	*C. parapsilopsis* ATCC 22019
C1	19.95 ± 0.64^{a}	22.21 ± 0.50^{a}	20.32 ± 0.44^{abc}	16.99 ± 0.35^{a}	16.03 ± 0.31^{ab}	21.09 ± 0.01^{a}	24.73 ± 0.41^{a}	16.00 ± 0.31^{ab}
C2	16.29 ± 0.37^{bcde}	20.93 ± 0.08^{b}	17.71 ± 0.35^{de}	14.78 ± 0.40^{bc}	13.88 ± 0.17^{cd}	17.38 ± 0.27^{bc}	22.00 ± 0.05^{bc}	15.55 ± 0.15^{abc}
A	15.52 ± 0.45^{bdf}	19.62 ± 0.26^{cd}	19.30 ± 0.25^{af}	13.46 ± 0.34^{d}	16.03 ± 0.24^{ab}	15.84 ± 0.37^{de}	22.07 ± 0.10^{bc}	14.49 ± 0.32^{cde}
S	12.49 ± 0.40^{g}	16.15 ± 0.49^{ef}	19.03 ± 0.60^{f}	11.43 ± 0.41^{e}	17.22 ± 0.02^{e}	19.97 ± 0.52^{a}	23.22 ± 0.31^{b}	20.75 ± 0.43^{f}
H	12.76 ± 0.35^{g}	15.45 ± 0.16^{e}	16.36 ± 0.22^{g}	11.51 ± 0.23^{e}	13.48 ± 0.13^{cd}	13.63 ± 0.25^{f}	15.18 ± 0.35^{d}	13.67 ± 0.61^{dg}
C1+C2	17.76 ± 0.13^{h}	18.54 ± 0.16^{g}	20.46 ± 0.25^{bc}	11.31 ± 0.30^{e}	13.09 ± 0.33^{cf}	17.14 ± 0.33^{b}	22.13 ± 0.25^{bc}	12.84 ± 0.20^{g}
C1+A	16.91 ± 0.34^{ch}	19.79 ± 0.43^{cd}	20.34 ± 0.25^{abc}	16.10 ± 0.93^{af}	14.28 ± 0.16^{d}	16.97 ± 0.14^{bd}	22.89 ± 0.29^{b}	13.31 ± 0.19^{dg}
C1+S	16.85 ± 0.22^{ceh}	19.80 ± 0.41^{cd}	19.20 ± 0.11^{f}	15.58 ± 0.24^{bf}	18.76 ± 0.08^{g}	18.35 ± 0.34^{cg}	22.73 ± 0.23^{b}	17.74 ± 0.61^{hi}
C1+H	16.79 ± 0.36^{ceh}	18.48 ± 0.32^{g}	21.24 ± 0.23^{b}	13.78 ± 0.45^{cd}	18.79 ± 0.31^{g}	18.08 ± 0.19^{bcg}	20.09 ± 0.52^{ef}	14.07 ± 0.69^{deg}
C2+A	16.38 ± 0.31^{bce}	20.52 ± 0.10^{bc}	21.00 ± 0.32^{b}	11.48 ± 0.10^{e}	15.51 ± 0.39^{a}	18.74 ± 0.10^{g}	22.33 ± 0.87^{bc}	13.90 ± 0.02^{deg}
C2+S	15.47 ± 0.38^{bdf}	19.05 ± 0.28^{dg}	18.51 ± 0.10^{dfh}	11.65 ± 0.59^{e}	15.56 ± 0.33^{a}	19.99 ± 0.38^{a}	21.21 ± 0.50^{ce}	18.20 ± 0.09^{h}
C2+H	15.96 ± 0.34^{bcde}	16.77 ± 0.36^{fh}	19.38 ± 0.08^{acf}	11.87 ± 0.29^{e}	12.38 ± 0.34^{f}	13.30 ± 0.65^{f}	19.78 ± 0.65^{ef}	13.25 ± 0.57^{dg}
A+S	14.59 ± 0.40^{f}	17.18 ± 0.37^{hi}	18.91 ± 0.41^{fh}	13.64 ± 0.26^{cd}	16.66 ± 0.33^{be}	17.80 ± 0.67^{bcg}	20.18 ± 0.68^{ef}	19.02 ± 0.16^{h}
A+H	15.80 ± 0.22^{bde}	18.12 ± 0.21^{gi}	16.84 ± 0.40^{eg}	14.05 ± 0.28^{cd}	14.17 ± 0.20^{d}	17.31 ± 0.21^{bc}	19.20 ± 0.71^{fg}	18.30 ± 0.56^{h}
S+H	15.30 ± 0.38^{bdf}	15.22 ± 0.26^{e}	17.39 ± 0.19^{eg}	10.65 ± 0.33^{e}	14.11 ± 0.56^{d}	17.92 ± 0.58^{bcg}	20.37 ± 0.51^{ef}	16.71 ± 0.37^{ai}
PAN	19.95 ± 0.64^{a}	22.21 ± 0.50^{a}	20.32 ± 0.44^{abc}	16.99 ± 0.35^{a}	16.03 ± 0.31^{ab}	21.09 ± 0.01^{a}	24.73 ± 0.41^{a}	16.00 ± 0.31^{ab}
Vancomycin (30 µg)	16.17 ± 0.26	17.21 ± 0.23	19.52 ± 0.52	15.73 ± 0.65	10.73 ± 0.20	21.87 ± 0.69	-	-
Fluconazole (10 µg)	-	-	-	-	-	-	33.09 ± 0.40	38.78 ± 0.98

Table S3. Antimicrobial activity of individual propolis ethanolic extracts (PEEs) and their binary combinations against Gram-negative bacterial strains, determined by the agar well diffusion assay, expressed in millimeters (mm). Data were expressed as the mean ± SD (n = 3). Different lowercase letters (a–h) in each column indicate significant differences, $p < 0.05$.

Treatment group	Gram- negative Strains							
	E. coli ATCC 8739	*E. coli* ATCC 25922	*P. aeruginosa* ATCC 27853	*K. pneumoniae* ATCC 13883	*P. mirabilis* ATCC 29906	*baumannii* ATCC 19606	*S. enterica* ATCC 25928	*S. typhimurium* ATCC 14028
C1	13.41 ± 0.40^{ab}	11.23 ± 0.27^{abc}	13.49 ± 0.35^{a}	12.57 ± 0.22^{a}	13.90 ± 0.25^{ab}	14.62 ± 0.27^{a}	12.09 ± 0.30^{ab}	13.19 ± 0.21^{ab}
C2	12.59 ± 0.29^{ac}	10.21 ± 0.25^{a}	12.60 ± 0.49^{abc}	11.09 ± 0.11^{bcd}	13.18 ± 0.14^{c}	14.97 ± 0.40^{a}	11.30 ± 0.30^{c}	13.45 ± 0.42^{abc}
A	11.92 ± 0.37^{cd}	11.07 ± 0.24^{ab}	12.55 ± 0.27^{bc}	10.48 ± 0.20^{bef}	14.40 ± 0.30^{a}	12.15 ± 0.26^{bc}	12.53 ± 0.29^{ade}	11.18 ± 0.05^{d}
S	11.20 ± 0.42^{d}	15.11 ± 0.38$_{d}$	14.67 ± 0.29^{d}	10.15 ± 0.13^{e}	19.36 ± 0.35^{d}	16.40 ± 0.55^{d}	21.15 ± 0.08^{f}	15.08 ± 0.19^{e}
H	12.34 ± 0.22^{acd}	11.27 ± 0.06^{abc}	12.63 ± 0.26^{abc}	11.41 ± 0.24^{cdg}	16.58 ± 0.12^{ef}	12.60 ± 0.29^{b}	12.96 ± 0.16^{deg}	12.71 ± 0.50^{af}

C1+C2	12.94 ± 0.62^{abc}	12.57 ± 0.16^{efgh}	13.23 ± 0.32^{ab}	10.28 ± 0.11^{ef}	11.28 ± 0.21^{g}	10.50 ± 0.28^{e}	12.37 ± 0.06^{abd}	10.01 ± 0.21^{g}
C1+A	12.00 ± 0.33^{cd}	12.23 ± 0.56^{cefg}	12.24 ± 0.33^{c}	11.37 ± 0.20^{cdg}	13.44 ± 0.20^{bc}	11.80 ± 0.22^{bc}	12.18 ± 0.21^{ab}	10.94 ± 0.03^{dg}
C1+S	13.99 ± 0.12^{be}	11.47 ± 0.21^{bce}	13.44 ± 0.20^{ab}	10.44 ± 0.26^{ef}	15.91 ± 0.19^{h}	14.94 ± 0.52^{a}	16.43 ± 0.23^{h}	14.36 ± 0.34^{ce}
C1+H	12.27 ± 0.27^{acd}	12.41 ± 0.28^{efgh}	12.03 ± 0.32^{c}	10.70 ± 0.24^{bef}	13.92 ± 0.09^{ab}	11.69 ± 0.53^{bc}	13.19 ± 0.21^{eg}	11.61 ± 0.12^{dh}
C2+A	12.97 ± 0.37^{abc}	12.88 ± 0.30^{fgh}	12.73 ± 0.25^{abc}	11.76 ± 0.24^{g}	11.97 ± 0.03^{i}	11.06 ± 0.47^{ce}	13.31 ± 0.24^{g}	11.42 ± 0.08^{dh}
C2+S	14.08 ± 0.31^{bef}	11.92 ± 0.75^{bcefg}	17.57 ± 0.36^{e}	11.47 ± 0.21^{cg}	16.05 ± 0.09^{eh}	14.86 ± 0.45^{a}	16.73 ± 0.25^{h}	12.71 ± 0.10^{af}
C2+H	12.27 ± 0.28^{acd}	11.78 ± 0.38^{bcef}	10.32 ± 0.18^{f}	10.80 ± 0.13^{bdf}	15.98 ± 0.18^{eh}	11.56 ± 0.26^{bce}	11.75 ± 0.34^{bc}	12.14 ± 0.39^{fh}
A+S	15.39 ± 0.31^{g}	13.37 ± 0.53^{h}	19.41 ± 0.24^{g}	11.37 ± 0.16^{cdg}	16.88 ± 0.18^{f}	16.80 ± 0.30^{d}	16.36 ± 0.19^{h}	13.44 ± 0.43^{abc}
A+H	13.03 ± 0.27^{abc}	11.47 ± 0.16^{bce}	12.12 ± 0.24^{c}	11.57 ± 0.25^{cg}	13.62 ± 0.27^{bc}	14.01 ± 0.28^{a}	16.72 ± 0.23^{h}	12.78 ± 0.65^{af}
S+H	14.87 ± 0.56^{efg}	11.90 ± 0.21^{bcefg}	15.53 ± 0.28^{d}	10.63 ± 0.13^{bef}	16.85 ± 0.26^{f}	12.45 ± 0.41^{b}	16.51 ± 0.45^{h}	13.77 ± 0.27^{bc}
PAN	13.41 ± 0.40^{ab}	11.23 ± 0.27^{abc}	13.49 ± 0.35^{a}	12.57 ± 0.22^{a}	13.90 ± 0.25^{ab}	14.62 ± 0.27^{a}	12.09 ± 0.30^{ab}	13.19 ± 0.21^{ab}
Gentamicin (30 µg)	16.83 ± 0.27	15.87 ± 0.27	17.27 ± 0.51	15.02 ± 0.16	20.85 ± 0.42	13.76 ± 0.10	16.55 ± 0.22	13.75 ± 0.35
