# Supplementary material for: Antioxidant and Antimicrobial Potential of Romanian Propolis Extract Combinations: Linking Chemical Composition with Biological Activity
Source: Antibiotics (Basel). 2026 Oct 1;15(10):972. doi: 10.3390/antibiotics15100972 (PMC13700676; doi:10.3390/antibiotics15100972)
Supplement: Supplementary file 1 [file antibiotics-15-00972-s001.zip › Supplementary_table 4 and 5_MIC.pdf]

Table S4. Minimum inhibitory concentration (MIC) values of individual propolis ethanolic extracts (PEEs) and their binary combinations against Gram-positive bacterial strains and yeasts.

	Gram-positive strains						Yeasts	
Condition	*S. aureus* MRSA ATCC 43300	*S. aureus* ATCC 25923	*S. epidermidis* ATCC 12228	*E. faecalis* ATCC 29212	*E. faecium* ATCC 19434	*L. monocytogenes* ATCC 35152	*C. albicans* ATCC 10231	*C. parapsilosis* ATCC 22019
C1	0.190	0.040	0.057	0.390	0.260	0.160	0.027	0.780
C2	0.780	0.120	0.320	0.650	1.170	0.320	0.040	0.780
A	0.780	0.190	0.190	2.600	0.390	0.390	0.090	0.780
S	2.080	0.450	0.190	4.160	0.520	0.260	0.040	0.090
H	2.600	0.650	0.390	3.120	1.300	2.080	0.780	2.080
C1+C2	0.390	0.160	0.090	4.160	2.080	0.320	0.073	1.560
C1+A	0.390	0.090	0.140	0.520	0.780	0.260	0.040	1.300
C1+S	0.520	0.190	0.260	1.040	0.190	0.320	0.033	0.650
C1+H	0.390	0.190	0.073	1.300	0.220	0.190	0.090	1.560
C2+A	0.390	0.110	0.090	3.120	0.910	0.190	0.073	1.560
C2+S	1.040	0.260	0.190	2.600	0.650	0.160	0.120	0.260
C2+H	0.520	0.320	0.260	4.160	2.080	1.560	0.160	2.080
A+S	0.650	0.260	0.190	1.560	0.390	0.390	0.073	0.190
A+H	1.040	0.160	0.390	1.300	0.650	0.650	0.260	0.260
S+H	0.780	0.650	0.390	6.250	0.780	0.390	0.190	0.520
PAN	1.040	0.190	0.390	0.780	1.560	0.780	0.390	0.910
Vancomycin (30 µg)	0.010	0.006	0.004	0.002	0.004	0.006	-	-
Fluconazole (10 µg)	-	-	-	-	-	-	0.003	0.003
Ethanol (80%)	> 50	> 50	> 50	> 50	> 50	> 50	> 50	> 50

MIC values are expressed in mg/mL

Table S5. Minimum inhibitory concentration (MIC) values of individual propolis ethanolic extracts (PEEs) and their binary combinations against Gram-negative bacterial strains and yeasts.

Condition	Gram-negative strains							
	E. coli ATCC 8739	*E. coli* ATCC 25922	*P. aeruginosa* ATCC 27853	*K. pneumoniae* ATCC 13883	*P. mirabilis* ATCC 29906	*A. baumannii* ATCC 19606	*S. enterica* ATCC 25928	*S. typhimurium* ATCC 14028
C1	1.560	2.600	1.560	1.560	0.780	0.650	1.560	0.780
C2	1.560	2.600	2.600	4.160	1.300	0.650	3.120	1.560
A	2.080	1.560	1.300	6.250	0.520	1.560	1.560	3.120
S	4.160	0.780	0.780	5.210	0.190	0.390	0.160	0.780
H	1.300	3.120	1.560	4.160	0.390	1.560	1.560	1.300
C1+C2	1.040	1.560	2.600	8.330	3.120	3.120	1.560	6.250
C1+A	3.120	1.560	1.560	2.600	1.560	2.600	1.300	3.120
C1+S	0.780	5.210	1.300	4.160	0.780	1.040	0.390	1.300
C1+H	1.040	1.560	2.080	2.600	1.040	2.600	1.560	3.120
C2+A	1.560	1.560	2.080	3.120	3.120	2.080	1.560	3.120
C2+S	0.780	3.120	0.390	3.120	0.650	0.650	0.390	2.080
C2+H	1.300	4.160	6.250	2.600	0.910	2.600	2.600	1.560
A+S	1.040	1.560	0.190	2.600	0.390	0.390	0.320	2.080
A+H	2.080	4.160	1.560	4.160	1.560	0.780	0.780	1.560
S+H	0.520	3.120	0.780	3.120	0.260	2.080	0.390	1.300
PAN	1.040	1.560	2.080	3.120	0.910	3.120	2.080	3.120
Gentamicin (30 μg)	0.020	0.020	0.006	0.006	0.010	0.006	0.010	0.002
Ethanol (80%)	> 50	> 50	> 50	> 50	> 50	> 50	> 50	> 50

MIC values are expressed in mg/mL.
