# Supplementary material for: Antioxidant and Antimicrobial Potential of Romanian Propolis Extract Combinations: Linking Chemical Composition with Biological Activity
Source: Antibiotics (Basel). 2026 Oct 1;15(10):972. doi: 10.3390/antibiotics15100972 (PMC13700676; doi:10.3390/antibiotics15100972)
Supplement: Supplementary file 1 [file antibiotics-15-00972-s001.zip › Supplementary_table 13 and 14 FIC.pdf]

Table S13. FICI values and interaction profiles of binary propolis ethanolic extract combinations against Gram-positive bacterial and yeast strains. Colour coding indicates the type of interaction.

	S. aureus MRSA ATCC 43300		*S. aureus* ATCC 25923		*S. epidermidis* ATCC 12228		*E. faecalis* ATCC 29212		*E. faecium* ATCC 19434		*L. monocytogenes* ATCC 35152		*C. albicans* ATCC 10231		*C. parapsilosis* ATCC 22019	
Condition	FICI	Int*	FICI	Int*	FICI	Int*	FICI	Int*	FICI	Int*	FICI	Int*	FICI	Int*	FICI	Int*
C1+C2	1.276	indifferent	2.667	indifferent	0.934	additive	8.533	antagonism	4.889	antagonism	1.500	indifferent	2.289	indifferent	2.000	indifferent
C1+A	1.276	indifferent	1.362	indifferent	1.603	indifferent	0.767	additive	2.500	indifferent	1.146	indifferent	0.971	additive	1.667	indifferent
C1+S	1.493	indifferent	2.586	indifferent	2.977	indifferent	1.458	indifferent	0.548	additive	1.615	indifferent	1.040	indifferent	4.028	antagonism
C1+H	1.101	indifferent	2.521	indifferent	0.740	additive	1.875	indifferent	0.508	additive	0.639	additive	1.743	indifferent	1.375	indifferent
C2+A	0.500	synergy	0.748	additive	0.377	synergy	3.000	indifferent	1.556	indifferent	0.540	additive	1.323	indifferent	2.000	indifferent
C2+S	0.917	additive	1.372	indifferent	0.797	additive	2.313	indifferent	0.903	additive	0.558	additive	3.000	indifferent	1.611	indifferent
C2+H	0.433	synergy	1.579	indifferent	0.740	additive	3.867	indifferent	1.689	indifferent	2.813	indifferent	2.103	indifferent	1.833	indifferent
A+S	0.573	additive	0.973	additive	1.000	indifferent	0.488	synergy	0.875	additive	1.250	indifferent	1.323	indifferent	1.177	indifferent
A+H	0.867	additive	0.544	additive	1.526	indifferent	0.458	synergy	1.083	indifferent	0.990	additive	1.611	indifferent	0.229	synergy
S+H	0.338	synergy	1.222	indifferent	1.526	indifferent	1.753	indifferent	1.050	indifferent	0.844	additive	2.497	indifferent	3.014	indifferent
PAN	1.808	indifferent	1.610	indifferent	2.640	indifferent	0.788	additive	3.107	indifferent	2.538	indifferent	7.788	antagonism	2.810	indifferent

Table S14. FICI values and interaction types of binary propolis ethanolic extract combinations against Gram-negative bacterial strains. Colour coding indicates the type of interaction.

	E. coli ATCC 8739		*E. coli* ATCC 25922		*P. aeruginosa* ATCC 27853		*K. pneumoniae* ATCC 13883		*P. mirabilis* ATCC 29906		*A. baumannii* ATCC 19606		*S. enterica* ATCC 25928		*S. typhimurium* ATCC 14028	
Condition	FICI	Int*	FICI	Int*	FICI	Int*	FICI	Int*	FICI	Int*	FICI	Int*	FICI	Int*	FICI	Int*
C1+C2	0.667	additive	0.600	additive	1.333	indifferent	3.671	indifferent	3.200	indifferent	4.800	antagonism	0.750	additive	6.010	antagonism
C1+A	1.750	indifferent	0.800	additive	1.100	indifferent	1.041	indifferent	2.500	indifferent	2.833	indifferent	0.833	additive	2.500	indifferent
C1+S	0.344	synergy	4.342	antagonism	1.250	indifferent	1.733	indifferent	2.553	indifferent	2.133	indifferent	1.344	indifferent	1.667	indifferent
C1+H	0.733	additive	0.550	additive	1.333	indifferent	1.146	indifferent	2.000	indifferent	2.833	indifferent	1.000	indifferent	3.200	indifferent
C2+A	0.875	additive	0.800	additive	1.200	indifferent	0.625	additive	4.200	antagonism	2.267	indifferent	0.750	additive	1.500	indifferent
C2+S	0.344	synergy	2.600	indifferent	0.325	synergy	0.674	additive	1.961	indifferent	1.333	indifferent	1.281	indifferent	2.000	indifferent
C2+H	0.917	additive	1.467	indifferent	3.205	indifferent	0.625	additive	1.517	indifferent	2.833	indifferent	1.250	indifferent	1.100	indifferent
A+S	0.375	synergy	1.500	indifferent	0.195	synergy	0.458	synergy	1.401	indifferent	0.625	additive	1.103	indifferent	1.667	indifferent
A+H	1.300	indifferent	2.000	indifferent	1.100	indifferent	0.833	additive	3.500	indifferent	0.500	synergy	0.500	synergy	0.850	additive
S+H	0.263	synergy	2.500	indifferent	0.750	additive	0.674	additive	1.018	indifferent	3.333	indifferent	1.344	indifferent	1.333	indifferent
PAN	0.577	additive	0.940	additive	1.547	indifferent	0.920	additive	2.148	indifferent	4.320	antagonism	3.533	indifferent	2.680	indifferent
