# Supplementary material for: Antioxidant and Antimicrobial Potential of Romanian Propolis Extract Combinations: Linking Chemical Composition with Biological Activity
Source: Antibiotics (Basel). 2026 Oct 1;15(10):972. doi: 10.3390/antibiotics15100972 (PMC13700676; doi:10.3390/antibiotics15100972)
Supplement: Supplementary file 1 [file antibiotics-15-00972-s001.zip › Supplementary_table1_HPLC.pdf]

Table S1. Phenolic profiles of the propolis ethanolic extracts. The data are expressed as the mean ± SD (n = 3). Different lowercase letters (a–c) in each column indicate significant differences $p < 0.05$.

Compound (μg/g)	Propolis extract ID															
	C1	C2	A	S	H	C1+C2	C1+A	C1+S	C1+H	C2+A	C2+S	C2+H	A+S	A+H	S+H	PAN
Apigenin	2.73 ± 0.16^{a}	16.04 ± 1.37^{abc}	16.41 ± 1.40^{abc}	13.02 ± 1.11^{abc}	4.54 ± 0.27^{ab}	27.39 ± 2.34^{abc}	32.10 ± 2.79^{c}	31.14 ± 2.68^{bc}	25.10 ± 2.15^{abc}	21.52 ± 1.84^{abc}	19.24 ± 1.65^{abc}	9.93 ± 0.60^{abc}	23.94 ± 2.05^{abc}	14.97 ± 1.28^{abc}	14.56 ± 1.24^{abc}	23.44 ± 2.01^{abc}
CAPE	113.39 ± 11.11^{abc}	157.69 ± 15.45^{abc}	0.17 ± 0.01^{c}	36.03 ± 3.08^{ac}	238.74 ± 23.39^{abc}	332.73 ± 32.60^{b}	195.19 ± 19.12^{abc}	191.19 ± 18.73^{abc}	248.24 ± 24.32^{ab}	114.43 ± 11.21^{abc}	107.27 ± 10.51^{abc}	206.65 ± 20.24^{abc}	187.57 ± 18.37^{abc}	62.32± 5.34^{abc}	185.31 ± 18.15^{abc}	-
Caffeicacid	44.79 ± 3.84^{ab}	69.47 ± 5.95^{ab}	79.63 ± 6.82^{ab}	21.95 ± 1.88^{a}	30.36 ± 2.60^{a}	96.83 ± 8.30^{ab}	172.17 ± 16.87^{b}	93.014 ± 7.974^{ab}	104.62 ± 10.25^{ab}	144.70 ± 14.17^{ab}	65.39 ± 5.60^{ab}	77.57 ± 6.65^{ab}	142.01 ± 13.91^{ab}	142.61 ± 13.97^{ab}	72.89 ± 6.24^{ab}	111.82 ± 10.95^{ab}
Catechin	-	-	-	-	1.51 ± 0.09^{a}	-	-	0.09 ± 0.01^{b}	-	0.19 ± 0.01^{ab}	0.24 ± 0.02^{ab}	0.17 ± 0.01^{ab}	-	-	-	-
Chlorogenic acid	-	-	-	0.75 ± 0.05^{ab}	-	-	1.01 ± 0.06^{ab}	-	0.60 ± 0.04^{a}	1.17 ± 0.07^{ab}	1.52 ± 0.09^{b}	1.01 ± 0.06^{ab}	-	-	-	-
Chrysin	198.13 ± 19.41^{abc}	190.71 ± 18.68^{abc}	159.11 ± 15.59^{abc}	32.44 ± 2.78^{abc}	5.37 ± 0.33^{a}	307.22 ± 30.10^{bc}	377.78 ± 37.02^{c}	243.73 ± 23.88^{abc}	229.49 ± 22.48^{abc}	260.46 ± 25.52^{abc}	133.29 ± 13.06^{abc}	110.13 ± 10.79^{abc}	201.60 ± 19.75^{abc}	167.99 ± 16.46^{abc}	28.75 ± 2.46^{ab}	204.04± 19.99^{abc}
Ferulic acid	33.08 ± 2.83^{a}	106.30 ± 10.41^{abc}	100.45 ± 9.84^{abc}	225.01 ± 22.04^{abc}	348.89 ± 34.18^{bc}	78.25 ± 6.71^{ac}	100.40 ± 9.83^{abc}	195.91 ± 19.19^{abc}	299.21 ± 29.31^{abc}	128.91 ± 12.63^{abc}	224.28 ± 21.97^{abc}	327.50 ± 32.08^{abc}	249.94 ± 24.48^{abc}	323.29 ± 31.67^{abc}	437.29 ± 42.84^{b}	247.30 ± 24.23^{abc}
Galangin	42.83 ± 3.67^{abc}	68.63 ± 5.88^{abc}	31.00 ± 2.65^{abc}	4.19 ± 0.25^{a}	92.03 ± 7.89^{bc}	107.15 ± 10.49^{c}	76.23 ± 6.53^{abc}	82.20 ± 7.05^{abc}	84.36 ± 7.23^{abc}	34.91 ± 2.99^{abc}	37.89 ± 3.24^{abc}	63.04 ± 5.40^{abc}	51.48 ± 4.41^{abc}	9.33 ± 0.57^{ab}	68.67 ± 5.88^{abc}	-
Kaempferol	8.087± 0.49^{ab}	-	-	-	13.86 ± 1.19^{b}	11.65 ± 0.99^{ab}	10.29 ± 0.88^{ab}	10.99 ± 0.94^{ab}	-	-	-	-	-	5.49 ± 0.34^{a}	-	-
Naringenin	62.11 ± 5.32^{a}	24.79 ± 2.12^{ab}	21.55 ± 1.84^{ab}	8.52 ± 0.52^{ab}	6.91 ± 0.42^{b}	44.74± 3.83^{ab}	37.17 ± 3.18^{ab}	28.99 ± 2.48^{ab}	34.21 ± 2.93^{ab}	47.29 ± 4.06^{ab}	7.83 ± 0.48^{ab}	6.67 ± 0.41^{b}	8.34 ± 0.51^{ab}	7.80 ± 0.48^{ab}	10.17 ± 0.87^{ab}	7.51 ± 0.46^{ab}
Naringin	1.43 ± 0.08^{a}	-	-	-	0.68 ± 0.04^{ab}	0.47 ± 0.02^{b}	0.70 ± 0.04^{ab}	1.27 ± 0.08^{ab}	-	-	-	-	-	-	-	-
Pinocembrin	391.44 ± 38.35^{a}	214.83 ± 21.05^{abc}	180.10 ± 17.64^{abc}	-	305.74 ± 29.95^{ab}	289.43 ± 28.36^{abc}	25.25 ± 2.16^{c}	236.93 ± 23.21^{bc}	200.47 ± 19.64^{abc}	115.21 ± 11.28^{abc}	134.55 ± 13.18^{abc}	114.53 ± 11.22^{abc}	73.59 ± 6.31^{bc}	180.67 ± 17.70^{abc}	-	-
Protocatechuic acid	1.29 ± 0.08^{a}	0.73 ± 0.04^{abc}	0.85 ± 0.05^{abc}	0.50 ± 0.03^{abc}	0.44 ± 0.03^{b}	1.00 ± 0.06^{abc}	1.05 ± 0.06^{ac}	0.86 ± 0.05^{abc}	0.83 ± 0.05^{abc}	0.79 ± 0.05^{abc}	0.60 ± 0.04^{abc}	0.59 ± 0.03^{abc}	0.67 ± 0.04^{abc}	0.57 ± 0.04^{abc}	0.45 ± 0.03^{bc}	0.74 ± 0.05^{abc}
Quercetin	-	-	-	-	-	10.90 ± 0.93^{a}	11.68 ± 1.00^{a}	9.87 ± 0.60^{ab}	9.17 ± 0.56^{ab}	5.86 ± 0.36^{ab}	3.76 ± 0.23^{b}	4.93 ± 0.30^{ab}	-	-	7.35 ± 0.45^{ab}	-
Quercitrin	19.69 ± 1.68^{a}	6.08 ± 0.37^{b}	-	-	-	-	-	-	-	-	-	-	-	-	-	-
Rosmarinic acid	1.228 ± 0.07^{abc}	0.96 ± 0.06^{c}	6.97 ± 0.42^{a}	2.80 ± 0.17^{abc}	2.19 ± 0.13^{abc}	1.003± 0.06^{bc}	5.32 ± 0.32^{abc}	2.38 ± 0.14^{abc}	1.72 ± 0.10^{abc}	6.01 ± 0.37^{abc}	3.04 ± 0.17^{abc}	4.39 ± 0.27^{abc}	3.10 ± 0.19^{abc}	6.69 ± 0.41^{ab}	3.91 ± 0.24^{abc}	-
Vanillic acid	1.34 ± 0.08^{a}	1.98 ± 0.12^{ab}	2.29 ± 0.14^{ab}	1.38 ± 0.08^{a}	1.93 ± 0.12^{ab}	2.70 ± 0.16^{ab}	3.39 ± 0.20^{ab}	2.78 ± 0.17^{ab}	2.97 ± 0.18^{ab}	3.82 ± 0.23^{ab}	3.12 ± 0.19^{ab}	3.63 ± 0.22^{ab}	3.72 ± 0.22^{ab}	4.09 ± 0.25^{b}	3.28 ± 0.20^{ab}	-
Vanillin	15.64 ± 1.34^{a}	24.83 ± 2.12^{abc}	49.72 ± 4.26^{abc}	41.88 ± 3.59^{abc}	38.61 ± 3.31^{abc}	22.75 ± 1.95^{ac}	43.05 ± 3.69^{abc}	38.10 ± 3.26^{abc}	35.55 ± 3.05^{abc}	45.17 ± 3.87^{abc}	40.33 ± 3.45^{abc}	37.88 ± 3.25^{abc}	61.53 ± 5.27^{b}	54.69 ± 4.69^{bc}	54.12 ± 4.64^{bc}	44.41 ± 3.81^{abc}
p-Coumaric acid	70.60 ± 6.05^{a}	143.60 ± 14.07^{ac}	156.57± 15.34^{abc}	206.85 ± 20.26^{abc}	323.64 ± 31.71^{abc}	152.68 ± 14.96^{abc}	260.01 ± 25.47^{abc}	320.29 ± 31.38^{abc}	490.21 ± 48.03^{abc}	251.65 ± 24.65^{abc}	312.74 ± 30.64^{abc}	483.35 ± 47.36^{abc}	420.22 ± 41.17^{abc}	545.14 ± 53.41^{bc}	629.28 ± 61.65^{b}	403.84 ± 39.56^{abc}
